# Conformational Landscapes and Energetics of Carbon Nanohoops and their Ring-in-Ring Complexes

**DOI:** 10.1021/acs.jpclett.4c01270

**Published:** 2024-06-24

**Authors:** Niklas Geue, Markus Freiberger, Stefan Frühwald, Andreas Görling, Thomas Drewello, Perdita E. Barran

**Affiliations:** 1 Michael Barber Centre for Collaborative Mass Spectrometry, Manchester Institute of Biotechnology, Department of Chemistry, The University of Manchester, 131 Princess Street, Manchester M1 7DN, U.K.; 2 Physical Chemistry I, Department of Chemistry and Pharmacy, Friedrich-Alexander-Universität Erlangen-Nürnberg, Egerlandstraße 3, 91058 Erlangen, Germany

## Abstract

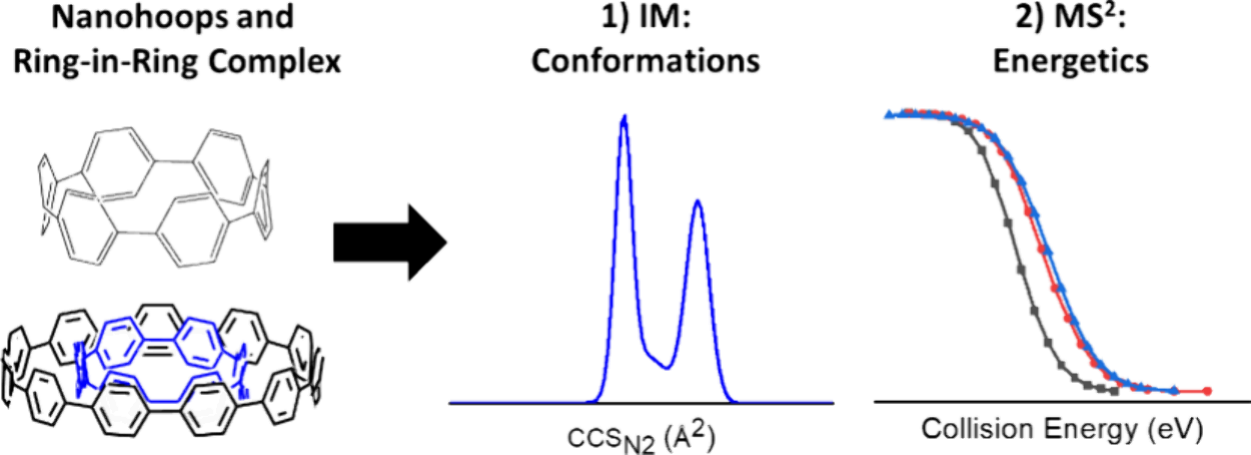

Carbon nanohoops are promising precursors for the synthesis of nanotubes, whose structural dynamics are not well understood. Here, we investigate the conformational landscape and energetics of cycloparaphenylenes (CPPs), a methylene-bridged CPP and a carbon nanobelt. These nanohoops can form host–guest complexes with other rings, and understanding their structure is crucial for predicting their properties and identifying potential applications. We used a combination of ion mobility, tandem mass spectrometry, and density functional theory to characterize the nanohoops and their ring-in-ring complexes, following the energetics and conformations of their disassembly from intact complexes to fragment ions. Our results show structural integrity of the nanohoops and host–guest complexes. They also reveal interesting trends in size, packing density, stability, and structure between [6]CPP, the methylene-bridged CPP, and the carbon nanobelt as guests in ring-in-ring complexes. Taken together, our work illustrates how mass spectrometry data can help to unravel the rules that govern the formation of carbon nanohoop assemblies.

Biological molecules are often highly flexible, and a diversity in structure is crucial for their function. Noncovalent compounds, whether synthesized or naturally found, exhibit bonds between different molecular units, and these noncovalent interactions are often weak and hence dynamic.^1^ This flexibility leads to diverse properties and functionalities, for example, in biological molecular machines, chaperone complexes, or DNA.^2^ Chemists have been mimicking such biological, noncovalent assemblies for decades by synthesizing supramolecules and analyzing their motions and dynamics.^3^ Their characterization can be challenging, due to their flexible and dynamic nature as well as difficulties of larger compounds in forming crystals.^1^

Modern mass spectrometry (MS) methods are uniquely placed to investigate supramolecules, in particular, since the invention of soft ionization methods such as electrospray ionization. MS offers a range of advantages, namely, the absence of solvent molecules and counterions in the gas phase, the capability to analyze complex mixtures, and the coupling to two-dimensional gas-phase techniques such as tandem mass spectrometry (MS^2^) and ion mobility (IM), which investigate their disassembly and stability (MS^2^) as well as their shape and size (IM), respectively.^4^ In MS^2^, ions are activated at user-defined kinetic energies, often via collisions with an inert gas (collision-induced dissociation, CID), and the ions undergo dissociation and/or structural rearrangements. IM also exploits gas collisions but at much weaker electric fields, and here collisions usually do not lead to fragmentation but to differences in the time the ions need to traverse the IM cell. This time can be converted to rotationally averaged collision cross section values (CCS), which inform on size and topology and can be compared to literature values and to those predicted computationally from candidate geometries. The use of MS to investigate such noncovalent assemblies has been regularly reviewed;^1,4−9^ however, in particular the simultaneous use of IM and MS^2^ for supramolecules has so far been underexplored.^4,10−13^ Such combined experiments, which are well-known for exploring the unfolding behavior of proteins similar to denaturation (“collisional unfolding”),^14^ can add structural information to our understanding of the disassembly process for dynamic supramolecules.

Cycloparaphenylenes (CPPs) are a class of carbon nanohoops, in which benzene rings are connected in *para*-position (Figure 1a center).^15−17^ They exhibit a concave cavity, which is a suitable binding site for convex, carbonaceous partners via π–π bonding, leading to host–guest complex formation with partners such as other CPPs,^18^ fullerenes,^19,20^ fullerene derivatives,^21,22^ or carbon nanobelts.^18^ CPPs can also be regarded as the smallest unit of carbon nanotubes, which are promising materials due to their high thermal conductivity and tensile strength. Hence, understanding the fundamental interactions in CPPs and their host–guest complexes is highly relevant to predict their reactivity and to understand the potential use of CPPs as a seed for carbon nanotube growth.

**Figure 1:**
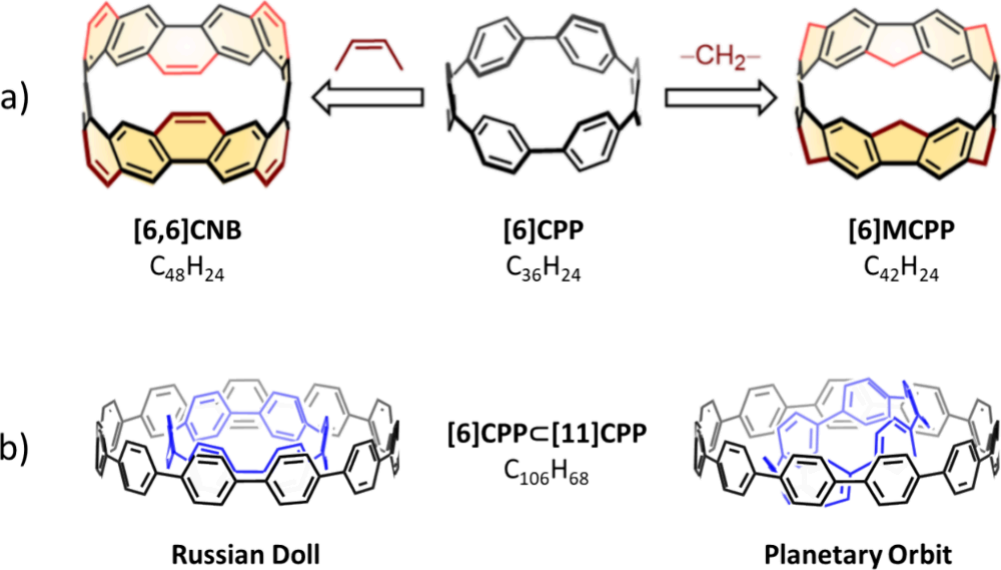
(a) Schematics of [6,6]CNB (*M* = 600 Da), [6]CPP (*M* = 456 Da), and [6]MCPP (*M* = 528 Da) including sum formulas. The differences in the bridging situation between the phenyl units are highlighted. The other [*n*]CPPs (not shown) are distinct to [6]CPP only in the number of repeating −C_6_H_4_– units. (b) Schematics of [6]CPP⊂[11]CPP (*M* = 1293 Da) in two conformations, “Russian foll” and “planetary orbit”. Reproduced from ref (25), Copyright 2022 American Chemical Society.

A major advantage of CPPs is the possibility to adapt their structure to form carbon nanohoops with varying ring strain, size, and aromaticity.^23^ Similar to [6]CPP, Itami and co-workers have synthesized a carbon nanobelt ([6,6]CNB, Figure 1a left), with −CH=CH– units between each of the phenyl rings,^24^ and a methylene-bridged cycloparaphenylene ([6]MCPP, Figure 1a right), where a methylene unit is added to each linker.^25^ While both [6]CPP and [6]MCPP are not fully conjugated, [6,6]CNB is (Figure 1a).

MS^2^ has been successfully employed to establish the noncovalent nature and the relative bond strengths of different carbon nanohoop-based host–guest complexes,^18,20,22,26−30^ and IM-MS was used to characterize CPP-based catenanes.^31^ The host–guest chemistry of carbon nanohoops is so far not well explored for cases where they act simultaneously as hosts and guests. [*n*]CPP⊂[*m*]CPP (with *n* < *m*; [*n*]CPP, guest; [*m*]CPP, host) complexes were synthesized for the first time by Hashimoto et al. in 2017,^32^ showing that a reaction mixture of different CPP sizes will selectively form [*n*]CPP⊂[*m*]CPP complexes with certain size differences between host CPP and guest CPP.^32,33^ The conformational landscape of [*n*]CPP⊂[*m*]CPP is debated, as Fomine et al. as well as Bachrat and Zayat had predicted such assemblies prior to synthesis, suggesting that either a “planetary orbit” or a “Russian doll” configuration is possible (Figure 1b).^34,35^ So far no direct experimental data support a conformational preference for a given [*n*]CPP⊂[*m*]CPP. Minameyer et al. used matrix-assisted laser desorption ionization mass spectrometry to analyze the MS^2^ behavior of [*n*]CPPs as well as the complex [5]CPP⊂[10]CPP,^36^ which like other [*n*]CPPs and [*n*]CPP⊂[*m*]CPP complexes readily form radical cations.^37^ Most recently, Freiberger et al. analyzed a range of [*n*]CPP⊂[*m*]CPP complexes using MS^2^, showing that complexes with phenyl unit differences of five or six are the most stable assemblies.^18^ The authors also showed that [*n*]CPP⊂[*m*]CPP complexes with similar size differences are usually more stable with increasing *n* and *m*, most likely due to stronger *π–π* interactions. Using the *π-*extended [6,6]CNB, they found a greater complex stability compared to the similarly sized [6]CPP as a guest, highlighting the potential of modifying the carbon nanohoop structure for improved properties.^18^

Here, we applied IM-MS, MS^2^ and density-functional theory (DFT) to understand the stability, conformational dynamics, and disassembly of [*n*]CPPs, the methylene-bridged [6]MCPP and the carbon nanobelt [6,6]CNB, both isolated and in their combined host–guest complexes. Our results show that all rings and ring-in-ring complexes maintain high structural integrity upon collisional activation, until the nanohoops dissociate and form various fragments including structures of higher CCS than the precursor. The ring-in-ring complexes revealed interesting trends in stability, size, structure, and packing density when varying the structures in the phenyl linkers between [6]CPP, [6]MCPP, and [6,6]CNB, unraveling the rules that govern the formation of such assemblies and yielding insights for the future design of carbon nanotubes and related materials. All experimental and computational details can be found in the Supporting Information.

We have studied the conformational landscape of [5]CPP-[12]CPP, as well as the methylated derivative [6]MCPP and the carbon nanobelt [6,6]CNB using IM-MS. Upon optimization of solution conditions, each nanohoop was transferred to the gas phase by nanoelectrospray ionization (nESI). The singly charged radical cation was typically found as the main species, although sometimes mixtures with the protonated ion were observed (Figure S1 for [11]CPP). As the radical ions dominated the spectrum, due to low ionization energies of carbon nanorings,^38^ they were used for all further investigations in this work. The collision cross section values in nitrogen (CCS_N2_) were determined (Table S1) and the DFT optimized structures were generated for all nanohoop radical cations (supplementary data set), from which in turn theoretical ^TH^CCS_N2_ values were simulated using the trajectory method of IMoS (Table S1).^39^ The agreement between theoretical and experimental CCS_N2_ values varied between 3 and 12%, depending on the nanohoop, with the theoretical value always being higher. This discrepancy is typical for small, supramolecular architectures (Figure S2 including discussion),^10^ and the overall agreement supports that the carbon rings are largely maintained in the gas phase.

We have previously shown that the relationship between CCS and mass of an ion is a suitable way to assess the packing density of atoms in synthetic ions,^4^ and we used the CCS/*m* slope to examine this property for the nanohoops (Figure 2). The regular [*n*]CPPs (*n* = 5–12) showed an almost perfect linear trend of a remarkably similar packing density, whereas both [6]MCPP and even more pronounced [6,6]CNB appear below this line, indicating a higher packing density. This is in agreement with a more dense structure based on the bridging situation ([6]MCPP, −CH_2_–; [6,6]CNB, −CH=CH−), compared to no bridges in [6]CPP (Figure 1).

**Figure 2:**
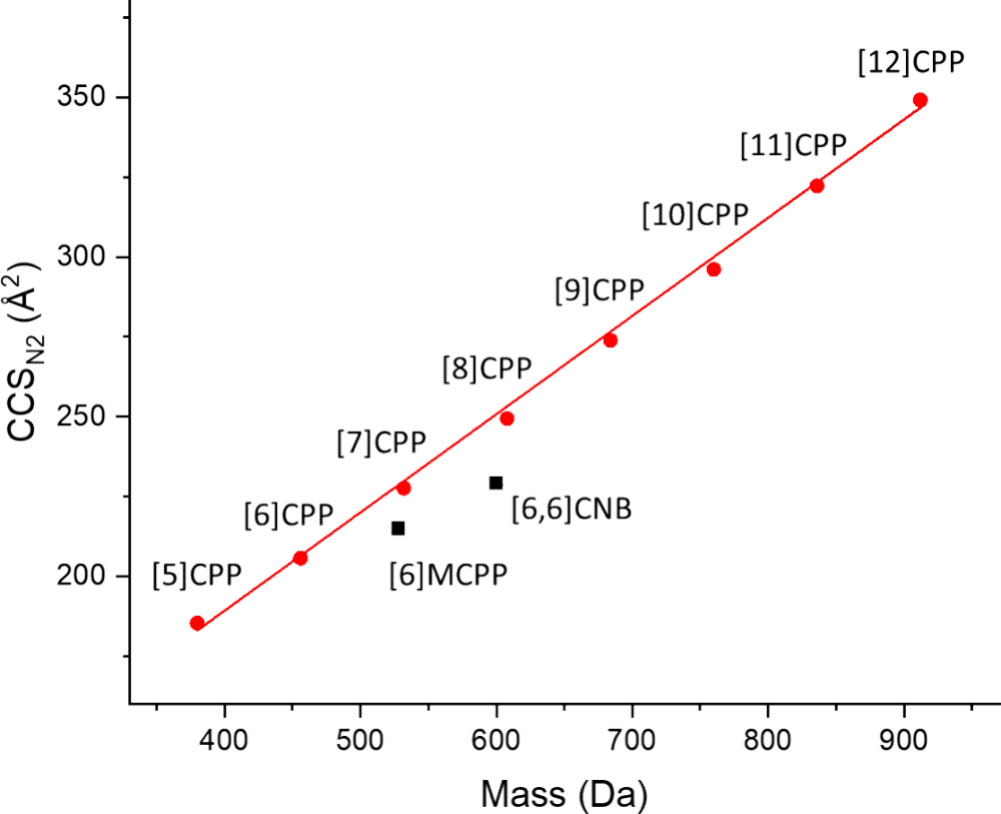
Experimental CCS_N2_ values of the isolated carbon nanohoop radical cations dependent on their mass. Error bars are shown but are smaller than symbol size. Red circles, regular [*n*]CPPs; black squares, [6]MCPP and [6,6]CNB. The data of the regular [*n*]CPPs were fitted and yielded CCS_N2_ = 66.28855 + 0.30754·mass (experimental; *R*^2^ = 0.99899).

In general, the three similarly shaped nanohoops [6]CPP, [6]MCPP, and [6,6]CNB yield a CCS_N2_ trend of [6,6]CNB > [6]MCPP > [6]CPP (Table S1), which is in agreement with their mass but not with their diameter *d* trend in the crystal structure [6,6]CNB (*d* = 8.3 Å) > [6]CPP (*d* = 8.1 Å) > [6]MCPP (*d* = 7.8 Å).^16,24,25^ While *d* is indicative of the nanohoop size, CCS measures the rotationally averaged cross section, and collisions inside the ring are also taken into account. Hence, the more strongly bridged species can have higher CCS_N2_ values.

Each nanohoop was *m*/*z*-selected with a quadrupole, predominantly as the radical cation, and activated at different collision energies using CID. As previously found for the fragmentation of CPP radical cations generated with laser desorption ionization, the tandem mass spectra of all nanohoops (including [6]MCPP and [6,6]CNB) show the subsequent dissociation of CH_*x*_ units and H atoms, reminiscent of polycyclic aromatic hydrocarbons (Figure S3 for [6]CPP).^36^ By measuring the ion mobility of the nanohoops upon collisional activation at user-defined energies, we were able to follow the conformational landscape of the activation process. For all nanohoops, a similar behavior was observed, although the larger rings required higher collision energies on average. The case of [6]CPP is shown in detail (Figure 3).

**Figure 3:**
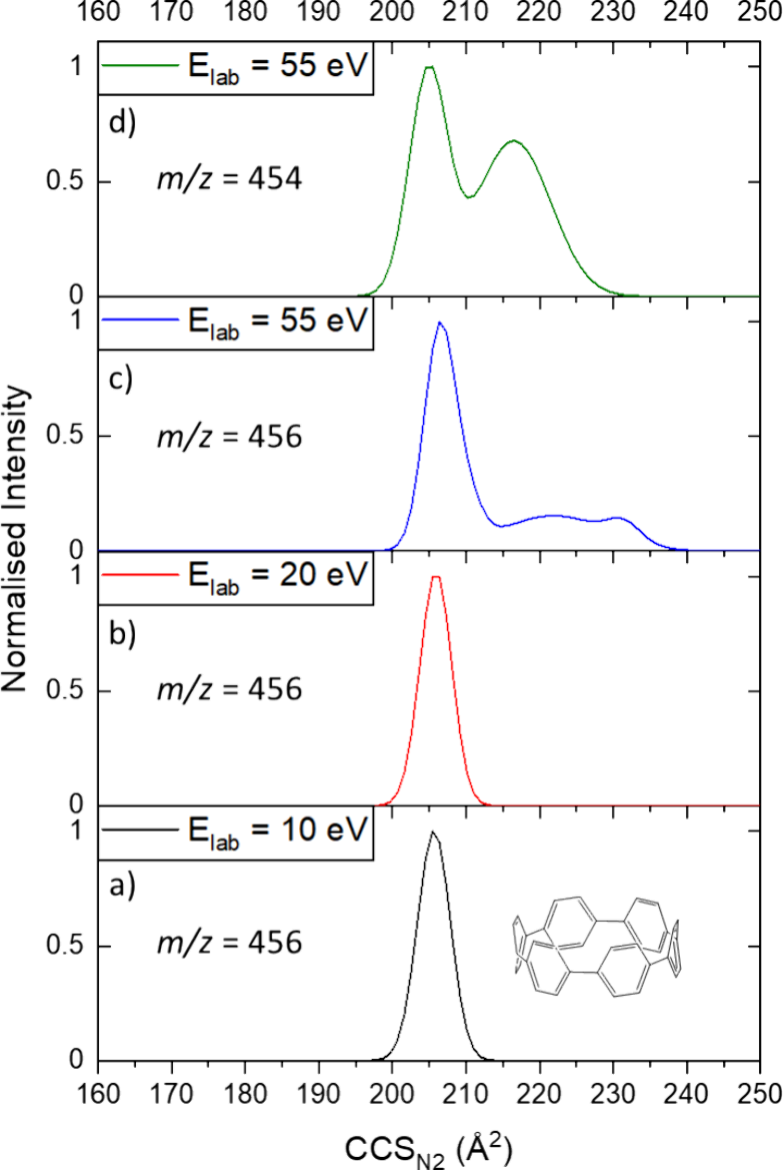
Activated IM-MS data of [6]CPP^+^. Collision cross section distributions (CCSD) of [6]CPP^+^ at *E*_lab_ of (a) 10 eV, (b) 20 eV, and (c) 55 eV. (d) CCSD of {[6]CPP – 2H}^+^ at *E*_lab_ = 55 eV. In (a), [6]CPP^+^ presents with a unimodal distribution centered around CCS_N2_ = 205.5 Å^2^, before a slight expansion to CCS_N2_ = 206.5 Å^2^ is observed in (b). At higher collision energies, fragmentation begins, and the *m*/*z*-selected [6]CPP^+^ extends to conformations up to CCS_N2_ ≈ 238 Å^2^. Similarly, the fragment that lost two hydrogen atoms (d) occupies a new conformation that is larger than the precursor (CCS_N2_ = 216 Å^2^).

When activating [6]CPP^+^ at low energies, a slight extension is observed (Figure 3a,b). This behavior occurs regularly, as shown for example in our previous studies on metallosupramolecular rings and rotaxanes.^10^ The ion remains remarkably rigid when ramping the collision energy further until fragmentation occurs and a range of fragments is observed that involve the loss of several CH_*x*_ units and/or single hydrogen atoms (Figure S3). At the same collision energies that lead to fragmentation, the parent ion [*n*]CPP^+^ extends partially with CCS_N2_ increases of up to 16% observed (Figure 3c). Similarly, the ions that remain with the same number of carbon atoms, but lose several hydrogen atoms, occupy conformations with higher CCS_N2_ than the parent ion (Figure 3d). Both observations are likely related to the cleavage of C–C bonds between the phenyl units in the rings, which would lead to a disruption of the ring structure and an extension. The fragments observed at lower *m*/*z* values show a rich conformational landscape (supplementary data set). It should be noted that [6]MCPP and [6,6]CNB show a qualitatively similar fragmentation and unfolding behavior, with [6]MCPP resulting in two particularly distinct parent ion conformations after the loss of two hydrogens (Figure S4). This behavior is somewhat surprising; however, the fragmentation and unfolding onset (in *E*_*lab*_) is significantly higher for [6]MCPP and [6,6]CNB than for [6]CPP (supplementary data set). This highlights the higher stability of the bridged nanohoops compared to CPPs.

We decided to investigate the differences in host–guest binding and conformations between [6]CPP, [6]MCPP, and [6,6]CNB, when they are encapsulated into the larger hosts [11]CPP and [12]CPP. These hosts were chosen as [*n*]CPP⊂[*m*]CPP^+^ complexes and showed the highest stability for a difference in phenyl units of 5 and 6.^18^ Equimolar solutions of all six combinations were prepared, and all intact host–guest complexes were transferred to the gas phase as radical cations using nESI. Their CCS_N2_ values were determined for ring-in-ring complexes with both hosts [11]CPP and [12]CPP, each yielding similar CCS_N2_ values for [6]CPP and [6]MCPP as well as slightly larger values for the [6,6]CNB assemblies (Table 1). This is in agreement with the larger size of the [6,6]CNB guest, which might contribute to collisions in the cavity and hence lead to an increased CCS_N2_ value.^24^ All six ring-in-ring complexes showed significantly higher packing densities than the isolated nanohoops, supporting the formation of host–guest complexes (Figure S5).

**Table 1:** Mass, CCS_N2_, *E*_50_, and Fragmentation Energies of the Six Ring-in-Ring Complexes^a^

ring-in-ring complex	mass (Da)	experimental CCS_N2_ (Å^2^)	*E*_50_ (eV)	fragmentation energy (DFT) (eV)
[6]CPP⊂[11]CPP	1293	355.8 ± 0.2	0.066^18^	2.03^18^ (PO)
[6]CPP⊂[12]CPP	1369	380.2 ± 0.3	0.064^18^	1.71 (PO)
[6,6]CNB⊂[11]CPP	1437	363.9 ± 0.6	0.078^18^	2.26^18^ (RD)
[6,6]CNB⊂[12]CPP	1513	388.3 ± 0.4	0.088	2.02 (RD)
[6]MCPP⊂[11]CPP	1364	353.7 ± 0.7	0.081	2.11 (RD)
[6]MCPP⊂[12]CPP	1441	383.4 ± 0.3	0.074	1.77 (RD)

a Data from our previous work is referenced.^18^ The fragmentation energies are based on the DFT calculations and are noted for the more stable of the two conformations (PO = planetary orbit; RD = Russian doll).

^TH^CCS_N2_ simulations were performed based on DFT calculations (Figures S6–S11) of the complexes in two different conformations, namely the “Russian doll” configuration as proposed by Fomine et al.,^34^ in which both rings are located in the same plane, and “planetary orbit” by Bachrach and Zayat,^35^ which has a ring–ring angle that is higher for more strained ring-in-ring assemblies (Figures 1b). We found no significant differences in ^TH^CCS_N2_ between the two conformations for any of the six host–guest assemblies (Table S2); the IM experiment supported by DFT and ^TH^CCS_N2_ simulations is not sufficient to resolve these structural differences. In respect to the guests, they follow the ^TH^CCS_N2_ order [6,6]CNB > [6]MCPP > [6]CPP for both hosts [11]CPP and [12]CPP, which largely agrees with the experimental CCS_N2_ trend.

We have further analyzed the stability of the six ring-in-ring carbon nanohoop complexes. As shown previously, these assemblies dissociate to the isolated host and guest rings (Figure 4 for [6]MCPP⊂[12]CPP), and the guest almost always retains the charge.^18^ Further fragmentation includes the disassembly of the isolated nanohoops, as discussed above. For the first dissociation step, survival yield curves (Figure 5) and *E*_50_ values were determined (Table 1), and the latter describes the energy at which 50% of the *m*/*z*-selected precursor ions dissociate to their fragments. As this value can be regarded as a relative measure of stability, this can indirectly inform on the conformational landscape and bonding situation in the nanohoop complexes.^40−42^ In our previous work, we had determined the *E*_50_ values for [6]CPP⊂[11]CPP, [6]CPP⊂[12]CPP, and [6,6]CNB⊂[11]CPP, and here we extend this study to the three other ring-in-ring complexes [6]MCPP⊂[11]CPP, [6]MCPP⊂[12]CPP, and [6,6]CNB⊂[12]CPP.^18^

**Figure 4:**
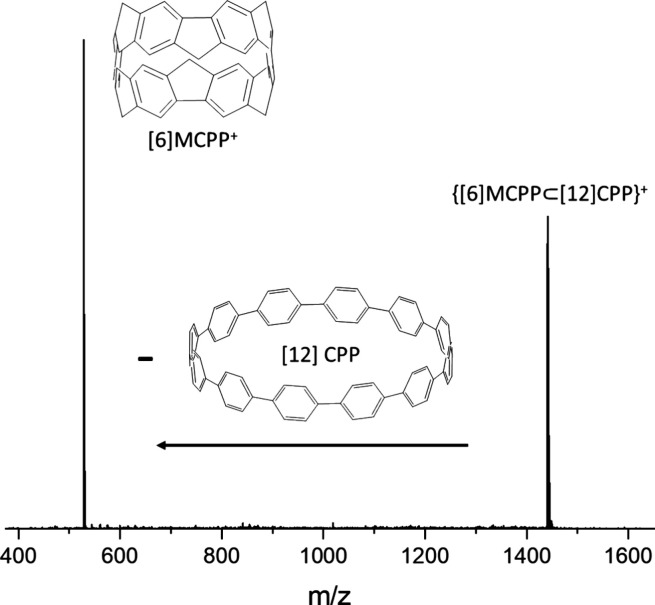
MS^2^ spectrum of {[6]MCPP⊂[12]CPP}^+^ (*m*/*z* = 1441) at *E*_lab_ = 20 eV. The dissociation of the ring-in-ring precursor follows the loss of the neutral host [12]CPP, resulting in the formation of the guest [6]MCPP^+^ radical cation.

**Figure 5:**
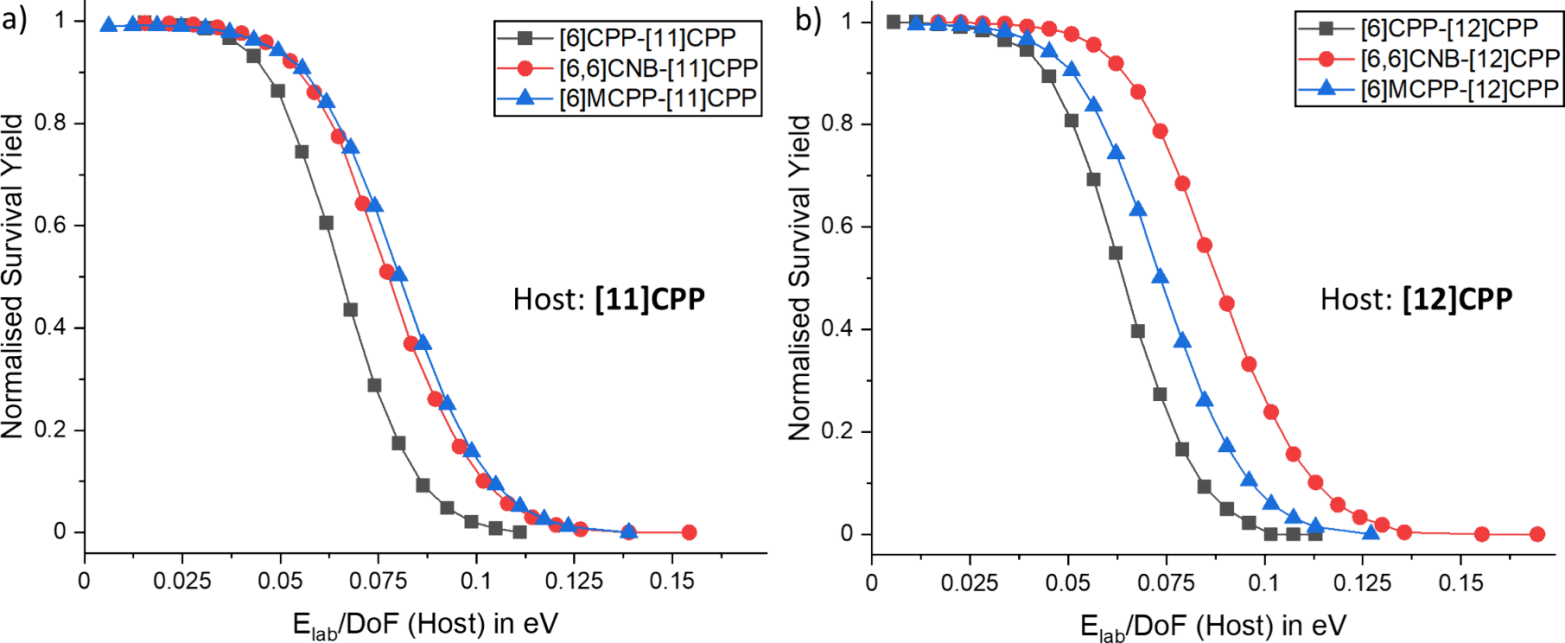
Survival yield curves for the studied ring-in-ring complexes with the hosts (a) [11]CPP and (b) [12]CPP. *E*_50_ values were determined and are noted in Table 1.

The data show that [11]CPP is the more suitable host for [6]CPP and [6]MCPP, whereas in the case of the nanobelt [6,6]CNB the host [12]CPP is preferred (Table 1). This observation is related to the large diameter *d* of [6,6]CNB, which is crucial for the π–π interactions between the host and guest. It was previously shown that a difference of five phenyl units is ideal for [*n*]CPP⊂[*m*]CPP complexes.^18,35^ Consequently, the enlarged structure of [6,6]CNB compared to [6]CPP as well as the increased number of interacting π-orbitals results in a higher stability with [12]CPP as the host. With regard to the guests, we found a stability trend of [6]MCPP > [6,6]CNB > [6]CPP for the host [11]CPP (Figure 5a), whereas the data for [12]CPP follow the order [6,6]CNB > [6]MCPP > [6]CPP (Figure 5b).

The disassembly follows the dissociation of the host from the charged guest (Figure 4), and hence, the interactions between both nanohoops determine the *E*_50_ values. As the carbon nanobelt has the largest π-system, their interactions with the host should be the strongest, and their ring-in-ring complexes can be expected to have the highest stability. This trend was indeed observed for the host [12]CPP, however for [11]CPP it is only the second most stable species. Based on the previously reported ideal size difference for ring-in-ring complexes, we assume that the carbon nanobelt is slightly too large for the host [11]CPP.^32,35^ This marginal size mismatch counteracts the increased π–π interactions and destabilizes the complex with [11]CPP.

The difference in stabilities between the [6]MCPP and [6]CPP complexes can also be explained by the difference in size. Since [6]MCPP is slightly smaller than [6]CPP, and [6]CPP is slightly too large for [11]CPP,^32,35^ the increased stability of [6]MCPP⊂[11]CPP over [6]CPP⊂[11]CPP is influenced by the size of the guest. There are also potentially more stabilizing interactions of the bridging hydrogens in [6]MCPP with the host π-system, which could slightly stabilize the complex.

Another possible explanation is different conformations of the host–guest assemblies. We compared the fragmentation energies of both “Russian doll” and “planetary orbit” (Figure 1b) for all complexes, revealing varying conformational preferences depending on the guest. While the fragmentation energies of the DFT optimized structures suggest that [6]CPP prefers the planetary orbit with both hosts [11]CPP and [12]CPP,^32,35^ the methylene-bridged species [6]MCPP forms more stable ring-in-ring complexes as a Russian doll (Table S2). This is indicative of a more favorable interaction landscape for [6]MCPP in the same plane as mentioned above and also a sign that hindered C–C rotations, due to the CH_2_ bridges, contribute to a better fit to [11]CPP and [12]CPP. Notably, the relative stabilities of the complexes based on the DFT fragmentation energies are in the order [6,6]CNB > [6]MCPP > [6]CPP with both hosts [11]CPP and [12]CPP, which is largely in agreement with the experimental trend.

The disassembly of the ring-in-ring complexes occurred at much lower energies than the dissociation of the single nanohoops, and following their conformational landscape upon collisional activation revealed highly rigid host–guest complexes that maintained their structure until fragmentation (Figure S12). The same applies to the newly formed guest cation ([6]CPP, [6]MCPP and [6,6]CNB), which showed no significant difference to the isolated nanohoops investigated in the section above (Figure S13). This suggests that the disassembly proceeds without the cleavage of any covalent bonds, and for all six complexes, the guest slips out of the host when exposed to collisional activation.

We suggest that the strong structural integrity of the carbon nanohoops and their host–guest complexes would make them interesting candidates for calibrating ion-mobility data of synthetic molecules,^4,43^ which is a necessity for most modern ion mobility experiments, namely, traveling-wave IM-MS,^44^ as used here, and trapped IM-MS.^45^ There is a lack of suitable calibrants for synthetic structures, as they should be easy to purchase/synthesize, rigid when exposed to high temperature and collisions, behave chemically similarly to the analyte, and cover similar *m*/*z* and CCS ranges. Although the *m*/*z* and CCS range of the structures investigated here is small, we believe that these and similar (larger) compounds have the potential to be used as calibrants in IM-MS. There is further a need to improve the correlation between CCS simulations based on DFT structures with experimental IM-MS data,^10^ and we believe that carbon nanohoops are suitable standards for this purpose.

We have demonstrated that the combination of IM-MS, MS^2^ and DFT calculations is highly suitable to characterize the dynamics of carbon nanohoops and their host–guest complexes. Our results show that both isolated nanohoops and their combined host–guest complexes are highly rigid when collisionally activated, although the rings can fragment to structures of higher CCS than the precursor. The findings of the different guests [6]CPP, [6]MCPP, and [6,6]CNB in ring-in-ring complexes with [11]CPP and [12]CPP showed interesting trends in CCS, packing density, fragmentation energy calculated with DFT and gas-phase stability, informing on the rules for the formation of such complexes based on their size, structure, and interactions. We believe that these results can inspire the design of novel, noncovalently bound carbon nanohoop complexes.

## Data Availability

Supplementary data set is available on Figshare https://figshare.com/articles/dataset/Supplementary_Dataset_for_Conformational_Dynamics_and_Energetics_of_Carbon_Nanohoops_and_their_Ring-in-Ring_Complexes_/25594068, which contains the raw data of ion mobility mass spectrometry and mass spectrometry measurements as well as the outputs from DFT calculations.

## Supporting Information Available

Mass spectra, CCS_N2_ values, DFT optimized structures, and collision cross section distributions (PDF)
Transparent Peer Review report available (PDF)

## References

[ref1] Geue N.; Winpenny R. E. P.; Barran P. E. Structural Characterisation Methods for Supramolecular Chemistry That Go beyond Crystallography. Chem. Soc. Rev. 2022, 51 (1), 8–27. 10.1039/D0CS01550D. 34817479

[ref2] Černý J.; Hobza P. Non-Covalent Interactions in Biomacromolecules. Phys. Chem. Chem. Phys. 2007, 9 (39), 5291–5303. 10.1039/b704781a. 17914464

[ref3] Coskun A.; Banaszak M.; Astumian R. D.; Stoddart J. F.; Grzybowski B. A. Great Expectations: Can Artificial Molecular Machines Deliver on Their Promise?. Chem. Soc. Rev. 2012, 41 (1), 19–30. 10.1039/C1CS15262A. 22116531

[ref4] Geue N.; Winpenny R. E. P.; Barran P. E. Ion Mobility Mass Spectrometry for Large Synthetic Molecules: Expanding the Analytical Toolbox. J. Am. Chem. Soc. 2024, 146 (13), 8800–8819. 10.1021/jacs.4c00354. 38498971 PMC10996010

[ref5] Cera L.; Schalley C. A. Supramolecular Reactivity in the Gas Phase: Investigating the Intrinsic Properties of Non-Covalent Complexes. Chem. Soc. Rev. 2014, 43 (6), 1800–1812. 10.1039/c3cs60360a. 24435245

[ref6] Lloyd Williams O. H.; Rijs N. J. Reaction Monitoring and Structural Characterisation of Coordination Driven Self-Assembled Systems by Ion Mobility-Mass Spectrometry. Front. Chem. 2021, 9, 682743 10.3389/fchem.2021.682743. 34169059 PMC8217442

[ref7] Wang H.; Guo C.; Li X. Multidimensional Mass Spectrometry Assisted Metallo-Supramolecular Chemistry. CCS Chem. 2022, 4 (3), 785–808. 10.31635/ccschem.021.202101408.

[ref8] Zimnicka M. M. Structural Studies of Supramolecular Complexes and Assemblies by Ion Mobility Mass Spectrometry. Mass Spectrom. Rev. 2024, 43 (3), 526–559. 10.1002/mas.21851. 37260128

[ref9] Geue N. Modern Electrospray Ionization Mass Spectrometry Techniques for the Characterization of Supramolecules and Coordination Compounds. Anal. Chem. 2024, 96 (19), 7332–7341. 10.1021/acs.analchem.4c01028. 38686955 PMC11099892

[ref10] Geue N.; Bennett T. S.; Arama A. A.; Ramakers L. A. I.; Whitehead G. F. S.; Timco G. A.; Armentrout P. B.; Mcinnes E. J. L.; Burton N. A.; Winpenny R. E. P.; Barran P. E. Disassembly Mechanisms and Energetics of Polymetallic Rings and Rotaxanes. J. Am. Chem. Soc. 2022, 144 (49), 22528–22539. 10.1021/jacs.2c07522. 36459680 PMC9756338

[ref11] Geue N.; Timco G. A.; Whitehead G. F. S.; McInnes E. J. L.; Burton N. A.; Winpenny R. E. P.; Barran P. E. Formation and Characterization of Polymetallic {CrxMy} Rings in Vacuo. Nat. Synth. 2023, 2 (10), 926–936. 10.1038/s44160-023-00383-7.

[ref12] Kruve A.; Caprice K.; Lavendomme R.; Wollschläger J. M.; Schoder S.; Schröder H. V.; Nitschke J. R.; Cougnon F. B. L.; Schalley C. A. Ion-Mobility Mass Spectrometry for the Rapid Determination of the Topology of Interlocked and Knotted Molecules. Angew. Chem., Int. Ed. 2019, 58 (33), 11324–11328. 10.1002/anie.201904541. 31173448

[ref13] Bell D. J.; Zhang T.; Geue N.; Rogers C. J.; Barran P. E.; Bowen A. M.; Natrajan L. S.; Riddell I. A. Hexanuclear Ln_6_L_6_ Complex Formation by Using an Unsymmetric Ligand. Chem.—Eur. J. 2023, 29 (71), e202302497 10.1002/chem.202302497. 37733973 PMC10946940

[ref14] Vallejo D. D.; Rojas Ramirez C.; Parson K. F.; Han Y.; Gadkari V. V.; Ruotolo B. T. Mass Spectrometry Methods for Measuring Protein Stability. Chem. Rev. 2022, 122 (8), 7690–7719. 10.1021/acs.chemrev.1c00857. 35316030 PMC9197173

[ref15] Jasti R.; Bhattacharjee J.; Neaton J. B.; Bertozzi C. R. Synthesis, Characterization, and Theory of [9]-, [12]-, and [18]Cycloparaphenylene: Carbon Nanohoop Structures. J. Am. Chem. Soc. 2008, 130 (52), 17646–17647. 10.1021/ja807126u. 19055403 PMC2709987

[ref16] Xia J.; Jasti R. Synthesis, Characterization, and Crystal Structure of [6]Cycloparaphenylene. Angew. Chem., Int. Ed. 2012, 51 (10), 2474–2476. 10.1002/anie.201108167. 22287256

[ref17] Lewis S. E. Cycloparaphenylenes and Related Nanohoops. Chem. Soc. Rev. 2015, 44 (8), 2221–2304. 10.1039/C4CS00366G. 25735813

[ref18] Freiberger M.; Frühwald S.; Minameyer M. B.; Görling A.; Drewello T. New Insights into Ring-In-Ring Complexes of [n]Cycloparaphenylenes Including the [12]Carbon Nanobelt. J. Phys. Chem. A 2023, 127 (45), 9495–9501. 10.1021/acs.jpca.3c05644. 37934505

[ref19] Iwamoto T.; Watanabe Y.; Sadahiro T.; Haino T.; Yamago S. Size-Selective Encapsulation of C60 by [10]Cycloparaphenylene: Formation of the Shortest Fullerene-Peapod. Angew. Chem., Int. Ed. 2011, 50 (36), 8342–8344. 10.1002/anie.201102302. 21770005

[ref20] Freiberger M.; Minameyer M. B.; Solymosi I.; Frühwald S.; Krug M.; Xu Y.; Hirsch A.; Clark T.; Guldi D. M.; von Delius M.; Amsharov K.; Görling A.; Pérez-Ojeda M. E.; Drewello T. Two Rings Around One Ball: Stability and Charge Localization of [1 : 1] and [2 : 1] Complex Ions of [10]CPP and C60/70[*]. Chem. – Eur. J. 2023, 29 (16), e202203734 10.1002/chem.202203734. 36507855

[ref21] Iwamoto T.; Slanina Z.; Mizorogi N.; Guo J.; Akasaka T.; Nagase S.; Takaya H.; Yasuda N.; Kato T.; Yamago S. Partial Charge Transfer in the Shortest Possible Metallofullerene Peapod, La@C82⊂[11]Cycloparaphenylene. Chem.—Eur. J. 2014, 20 (44), 14403–14409. 10.1002/chem.201403879. 25224281

[ref22] Freiberger M.; Solymosi I.; Freiberger E. M.; Hirsch A.; Pérez-Ojeda M. E.; Drewello T. A Molecular Popeye: Li+@C60 and Its Complexes with [n]Cycloparaphenylenes. Nanoscale 2023, 15 (12), 5665–5670. 10.1039/D2NR07166E. 36896752

[ref23] Li Y.; Kono H.; Maekawa T.; Segawa Y.; Yagi A.; Itami K. Chemical Synthesis of Carbon Nanorings and Nanobelts. Acc. Mater. Res. 2021, 2 (8), 681–691. 10.1021/accountsmr.1c00105.

[ref24] Povie G.; Segawa Y.; Nishihara T.; Miyauchi Y.; Itami K. Synthesis of a Carbon Nanobelt. Science 2017, 356 (6334), 172–175. 10.1126/science.aam8158. 28408599

[ref25] Li Y.; Segawa Y.; Yagi A.; Itami K. A Nonalternant Aromatic Belt: Methylene-Bridged [6]Cycloparaphenylene Synthesized from Pillar[6]Arene. J. Am. Chem. Soc. 2020, 142 (29), 12850–12856. 10.1021/jacs.0c06007. 32603101

[ref26] Xu Y.; Kaur R.; Wang B.; Minameyer M. B.; Gsänger S.; Meyer B.; Drewello T.; Guldi D. M.; von Delius M. Concave–Convex π–π Template Approach Enables the Synthesis of [10]Cycloparaphenylene–Fullerene [2]Rotaxanes. J. Am. Chem. Soc. 2018, 140 (41), 13413–13420. 10.1021/jacs.8b08244. 30234982

[ref27] Xu Y.; Wang B.; Kaur R.; Minameyer M. B.; Bothe M.; Drewello T.; Guldi D. M.; von Delius M. A Supramolecular [10]CPP Junction Enables Efficient Electron Transfer in Modular Porphyrin–[10]CPP⊃Fullerene Complexes. Angew. Chem., Int. Ed. 2018, 57 (36), 11549–11553. 10.1002/anie.201802443. 29985554

[ref28] Xu Y.; Gsänger S.; Minameyer M. B.; Imaz I.; Maspoch D.; Shyshov O.; Schwer F.; Ribas X.; Drewello T.; Meyer B.; von Delius M. Highly Strained, Radially π-Conjugated Porphyrinylene Nanohoops. J. Am. Chem. Soc. 2019, 141 (46), 18500–18507. 10.1021/jacs.9b08584. 31710474

[ref29] Schwer F.; Zank S.; Freiberger M.; Kaur R.; Frühwald S.; Robertson C. C.; Görling A.; Drewello T.; Guldi D. M.; von Delius M. Synthesis and C60 Binding of Aza[10]CPP and N-Methylaza[10]CPP. Org. Mater. 2022, 4 (2), 7–17. 10.1055/a-1814-7686.

[ref30] Schaub T. A.; Zieleniewska A.; Kaur R.; Minameyer M.; Yang W.; Schüßlbauer C. M.; Zhang L.; Freiberger M.; Zakharov L. N.; Drewello T.; Dral P. O.; Guldi D. M.; Jasti R. Tunable Macrocyclic Polyparaphenylene Nanolassos via Copper-Free Click Chemistry. Chem.–Eur. J. 2023, 29 (33), e202300668 10.1002/chem.202300668. 36880222

[ref31] Zhang W.; Abdulkarim A.; Golling F. E.; Räder H. J.; Müllen K. Cycloparaphenylenes and Their Catenanes: Complex Macrocycles Unveiled by Ion Mobility Mass Spectrometry. Angew. Chem., Int. Ed. 2017, 56 (10), 2645–2648. 10.1002/anie.201611943. 28146311

[ref32] Hashimoto S.; Iwamoto T.; Kurachi D.; Kayahara E.; Yamago S. Shortest Double-Walled Carbon Nanotubes Composed of Cycloparaphenylenes. ChemPlusChem 2017, 82 (7), 1015–1020. 10.1002/cplu.201700097. 31961607

[ref33] Zhao C.; Liu F.; Feng L.; Nie M.; Lu Y.; Zhang J.; Wang C.; Wang T. Construction of a Double-Walled Carbon Nanoring. Nanoscale 2021, 13 (9), 4880–4886. 10.1039/D0NR08931A. 33625431

[ref34] Fomine S.; Zolotukhin M. G.; Guadarrama P. “Russian Doll” Complexes of [n]Cycloparaphenylenes: A Theoretical Study. J. Mol. Model. 2012, 18 (9), 4025–4032. 10.1007/s00894-012-1402-7. 22460523

[ref35] Bachrach S. M.; Zayat Z.-C. “Planetary Orbit” Systems Composed of Cycloparaphenylenes. J. Org. Chem. 2016, 81 (11), 4559–4565. 10.1021/acs.joc.6b00339. 27163409

[ref36] Minameyer M. B.; Xu Y.; Frühwald S.; Görling A.; von Delius M.; Drewello T. Investigation of Cycloparaphenylenes (CPPs) and Their Noncovalent Ring-in-Ring and Fullerene-in-Ring Complexes by (Matrix-Assisted) Laser Desorption/Ionization and Density Functional Theory. Chem.–Eur. J. 2020, 26 (40), 8729–8741. 10.1002/chem.202001503. 32476186 PMC7497255

[ref37] Kayahara E.; Fukayama K.; Nishinaga T.; Yamago S. Size Dependence of [n]Cycloparaphenylenes (N=5–12) in Electrochemical Oxidation. Chem.–Asian J. 2016, 11 (12), 1793–1797. 10.1002/asia.201600582. 27137132

[ref38] Wong B. M. Optoelectronic Properties of Carbon Nanorings: Excitonic Effects from Time-Dependent Density Functional Theory. J. Phys. Chem. C 2009, 113 (52), 21921–21927. 10.1021/jp9074674. PMC3317592 22481999

[ref39] Shrivastav V.; Nahin M.; Hogan C. J.; Larriba-Andaluz C. Benchmark Comparison for a Multi-Processing Ion Mobility Calculator in the Free Molecular Regime. J. Am. Soc. Mass Spectrom. 2017, 28 (8), 1540–1551. 10.1007/s13361-017-1661-8. 28477243

[ref40] Kertesz T. M.; Hall L. H.; Hill D. W.; Grant D. F. CE50: Quantifying Collision Induced Dissociation Energy for Small Molecule Characterization and Identification. J. Am. Soc. Mass Spectrom. 2009, 20 (9), 1759–1767. 10.1016/j.jasms.2009.06.002. 19616966

[ref41] Hill D. W.; Baveghems C. L.; Albaugh D. R.; Kormos T. M.; Lai S.; Ng H. K.; Grant D. F. Correlation of Ecom 50 Values between Mass Spectrometers: Effect of Collision Cell Radiofrequency Voltage on Calculated Survival Yield. Rapid Commun. Mass Spectrom. 2012, 26 (19), 2303–2310. 10.1002/rcm.6353. 22956322 PMC3439163

[ref42] Chakraborty P.; Baksi A.; Khatun E.; Nag A.; Ghosh A.; Pradeep T. Dissociation of Gas Phase Ions of Atomically Precise Silver Clusters Reflects Their Solution Phase Stability. J. Phys. Chem. C 2017, 121 (20), 10971–10981. 10.1021/acs.jpcc.6b12485.

[ref43] Benoit F.; Wang X.; Dai J.; Geue N.; England R. M.; Bristow A. W. T.; Barran P. E. Exploring the Conformational Landscape of Poly(L-Lysine) Dendrimers Using Ion Mobility Mass Spectrometry. Anal. Chem. 2024, 96, 9390–9398. 10.1021/acs.analchem.4c00099 38812282 PMC11170554

[ref44] Shvartsburg A. A.; Smith R. D. Fundamentals of Traveling Wave Ion Mobility Spectrometry. Anal. Chem. 2008, 80 (24), 9689–9699. 10.1021/ac8016295. 18986171 PMC2761765

[ref45] Michelmann K.; Silveira J. A.; Ridgeway M. E.; Park M. A. Fundamentals of Trapped Ion Mobility Spectrometry. J. Am. Soc. Mass Spectrom. 2015, 26 (1), 14–24. 10.1007/s13361-014-0999-4. 25331153

