# Supplementary material for: Conformational Landscapes and Energetics of Carbon Nanohoops and their Ring-in-Ring Complexes
Source: J Phys Chem Lett. 2024 Jun 24;15(26):6805–11. doi: 10.1021/acs.jpclett.4c01270 (PMC11229059; doi:10.1021/acs.jpclett.4c01270)
Supplement: Supplementary file 1 — jz4c01270_si_001.pdf [file jz4c01270_si_001.pdf]

Supporting Information

Conformational Landscapes and Energetics of Carbon Nanohoops and their Ring-In-Ring Complexes

Niklas Geue[1,†], Markus Freiberger[2,†], Stefan Frühwald[2], Andreas Görling[2], Thomas Drewello[2,*] and Perdita E. Barran[1,*]

[1]Michael Barber Centre for Collaborative Mass Spectrometry, Manchester Institute of Biotechnology, Department of Chemistry, The University of Manchester, 131 Princess Street, Manchester, M1 7DN, UK. [2]Physical Chemistry I, Department of Chemistry and Pharmacy, Friedrich-Alexander-Universität Erlangen-Nürnberg, Egerlandstraße 3, 91058 Erlangen, Germany.

[*]Corresponding Authors: thomas.drewello@fau.de, perdita.barran@manchester.ac.uk

[†]These authors contributed equally.

Table of Contents

Methods.......... 3

Figure S1: Mass spectrum of [11]CPP 6

Table S1: Experimental and theoretical CCS_{N2} values of the isolated carbon nanohoop radical cations. 7

Figure S2: Experimental and theoretical CCS_{N2} values of the isolated carbon nanohoop radical cations in dependence of their mass. 8

Figure S3: MS^2 spectra of $[6]CPP^+$ at different collision energies. 10

Figure S4: Collision cross section distribution of $\{[6]MCPP – 2 H\}^+$ at E_{lab} = 80 eV. 11

Figure S5: Experimental CCS_{N2} values of the isolated carbon nanohoops and ring-in-ring complexes in dependence of their mass. 12

Figure S6: DFT optimised structures of [6]CPP⊂[11]CPP 13

Figure S7: DFT optimised structures of [6]CPP⊂[12]CPP 14

Figure S8: DFT optimised structures of [6,6]CNB⊂[11]CPP.......... 15

Figure S10: DFT optimised structures of [6]MCPP⊂[11]CPP.......... 17

Figure S11: DFT optimised structures of [6]MCPP⊂[12]CPP.......... 18

Table S2: Experimental CCS_{N2} values as well as theoretical CCS_{N2} values and fragmentation energies of ring-in-ring complexes in two different conformations 19

Figure S12: Collision cross section distributions of $\{[6]MCPP⊂[12]CPP\}^+$ 20

Figure S13: Collision cross section distributions of $[6]MCPP^+$ 21

References 22

Methods

Sample Preparation

All reagents and solvents were purchased from TCI, VWR or Merck and used without further purification. The nanohoop stock solutions were prepared in DCM (0.2 - 0.5 g L^{-1}). For the ion mobility mass spectrometry experiments, the analyte solutions consisted of equimolar amounts of the nanohoops at a concentration of 10^{-4} mol L^{-1}. To achieve stable spraying conditions, a solvent mixture of MeOH and tol (1:1, v:v) was chosen, to which a small amount of TFA was added. For the tandem mass spectrometry experiments including the determination of E_{50} values, analyte solutions were prepared by mixing equimolar amounts of the stock solutions in a mixture of ACN/DCM/toluene (3:2:1, v:v) with a final concentration of 10^{-5} mol L^{-1}. A small amount of TFA was added to enhance radical cation formation when needed.

Ion Mobility Mass Spectrometry

Samples were ionized and transferred to the gas phase with a nESI source and were sprayed from borosilicate glass capillaries (World Precision Instruments). The latter were pulled on the Flaming/Brown P-2000 laser puller (Sutter Instrument Company). The capillary voltage (typically 1.0 – 1.5 kV) was applied through a platinum wire (diameter 0.125 mm, Goodfellow) inserted into the nESI capillaries. Source temperatures of 50 °C were applied.

IM–MS experiments were performed on a Synapt G2 (Waters Corp). Following ionization (cone voltage, typically 50 – 100 V; purge gas flow: 500 L h^{-1}), ions were transferred to the trap and activated (trap voltage: 0 – 190 V), if appropriate, and further injected to the travelling wave drift cell (Synapt). They were separated by using a non-uniform electric field under a constant nitrogen gas flow (75 mL min^{-1}) with travelling waves (wave height: 40 V; velocity: 550 – 700 m s^{-1}), pushing the ions through the drift cell or ring, respectively. Lastly, they were transferred (transfer voltage: 4 V) to a time-of-flight mass analyser.

Experimentally obtained arrival times and their distributions were converted to collisional cross-sections and their distributions via published calibration procedures.[1] The Agilent tune mix was used as a calibrant.[2]

Tandem Mass Spectrometry and E_{50} values

MS^2 experiments for breakdown curves and E_{50} values were performed using a quadrupole time-of-flight mass spectrometer (micrOToF-Q II; Bruker Daltonics, Bremen, Germany) equipped with an ESI source. The sample solutions were directly injected into the ESI source via a syringe pump at a flow rate of 180 µL h^{-1}. Between the needle and the capillary entrance, a voltage of − 4.5 kV was applied. The instrument parameters were optimized to obtain good intensities for each experiment. N_2 was used as collision gas provided by a Parker LCMS64 nitrogen generator with a purity of 99.999% and a flow rate of 0.2 L min^{-1}.

The survival yield (*SY*) is measured and calculated for different collision energies, and represents the ratio of precursor ions that survive the collision event relative to the total of all ions observed. The collision energy is defined as the laboratory energy (E_{lab}) divided by the degrees of freedom (*DoF*) of the host nanohoop. This unconventional energy scale was chosen based on our previous publications,[3,4] and following the assumption that the guest nanohoop is shielded from collisions as it is encapsulated inside the pocket of the host nanohoop. Therefore, most collisions occur between the collision gas and the host. Dividing E_{lab} by *DoF* compensates for the difference in *DoF* of the precursor ions, which makes their direct comparison possible. The *DoF* were calculated according to Equation S1:

$$DOF = 3n - 6 \qquad \text{(Eq. S1)}$$

with *n* being the number of atoms in the host CPP.

The *SY* curves were measured under multiple collision conditions and fitted with a sigmoid Boltzmann function. The collision energy E_{50}, at which 50% of the complex has dissociated into its fragments, is used as a relative measure of its stability.[5,6]

Computational Details

Calculations using density-functional theory (DFT) were performed with the TURBOMOLE program package (Version 7.2)[7] on the B3LYP/def2-TZVP level of theory.[8–10] As supramolecular host-guest complexes between carbon nanohoops depend on Van-der-Waals (VdW) interactions, the D3 dispersion correction[11] was employed. Natural population

analyses[12] were conducted for the calculation of net charges on each atom. The program NCIPLOT (Version 3.0)[13] was used to visualize the areas of non-covalent interactions. Regions of non-covalent interactions were identified by the calculation of the reduced density gradient (RDG), which is given by Equation S2:

$$RDG = \frac{1}{2\,(3\pi^2)^{1/3}}\frac{|\nabla\rho|}{\rho^{4/3}} \qquad \text{(Eq. S2)}$$

The RDG can distinguish between regions far away from the nuclei, where the value of the RDG is large and regions of non-covalent interactions, where the RDG approaches 0. For visualization of long-range interactions contour plots of the RDG at low electron density (ρ = 0.2 was chosen as threshold) are calculated. The second largest eigenvalue of the electron ρ density Hessian matrix can be used to identify attractive or repulsive interactions as it changes its value accordingly in the expression sign(λ_2)ρ. For visualizations of non-covalent interactions, isosurfaces of the RDG at 0.3 a.u. and a color scale of $-0.05 < \rho < 0.05$ were selected.

Fragmentation energies were calculated as energy differences between the energies of the individual nanohoops and the energies of the ring-in-ring complex radical cations.

Theoretical *CCS* values in nitrogen gas ($^{TH}CCS_{N2}$, TH: theoretical) were obtained from the software IMoS by using the trajectory method in nitrogen gas including quadrupole potential (number of orientations 3, gas molecules per orientation 300,000, temperature 298 K, and pressure 101,325 Pa = 1 atm).[14]

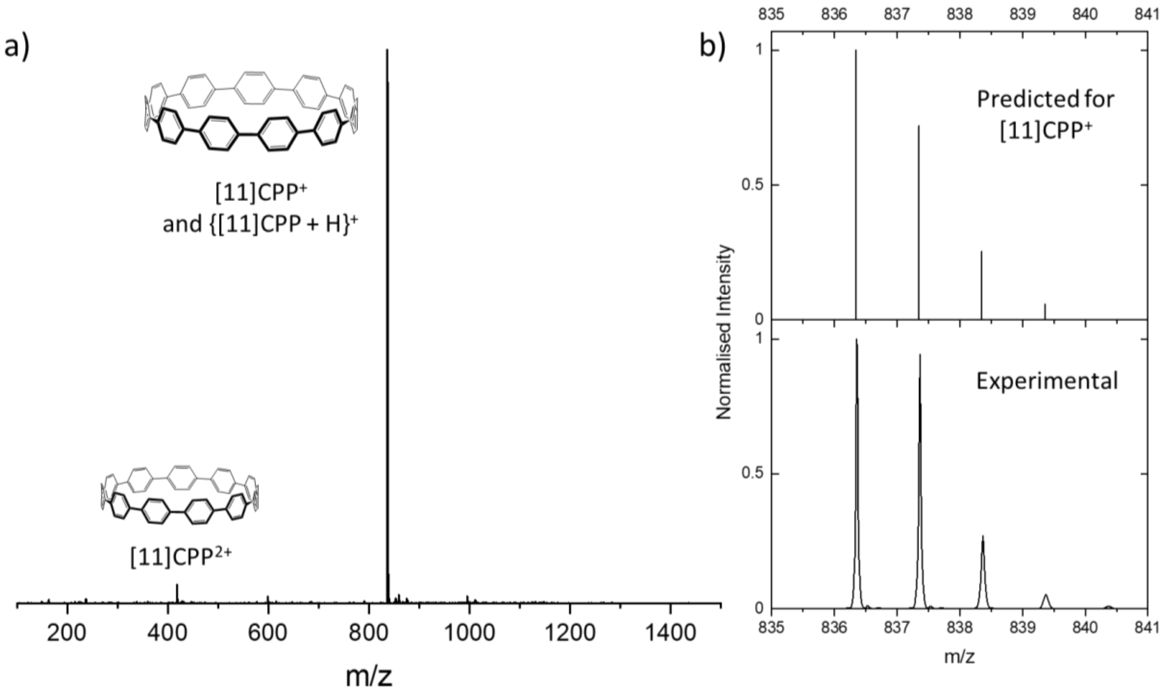

Figure S1: Mass spectrum of [11]CPP. a) Full MS shows the presence of the singly charged radical cation $[11]CPP^{+}$ with traces of the protonated species $\{[11]CPP + H\}^{+}$ as well as the doubly charged $[11]CPP^{2+}$. b) Comparison of experimental isotopic distribution and the one predicted for $[11]CPP^{+}$. The increased experimental intensity for the monoisotopic peak at 837.4 *m/z* suggests a mixture of the radical cation with the protomer.

Table S1: Experimental and theoretical CCS_{N2} values of the isolated carbon nanohoop radical cations.

Carbon Nanohoop	*m/z*	Experimental CCS_{N2} (Å^2)	Theoretical $^{TH}CCS_{N2}$ (Å^2)
[5]CPP	380	185.4 ± 0.1	196.1 ± 0.8
[6]CPP	456	205.7 ± 0.1	219.2 ± 0.4
[7]CPP	532	227.6 ± 0.2	246.1 ± 0.3
[8]CPP	608	249.4 ± 0.2	274.3 ± 0.5
[9]CPP	684	273.9 ± 0.2	303.9 ± 0.8
[10]CPP	760	296.1 ± 0.3	331.8 ± 0.8
[11]CPP	836	322.3 ± 0.1	362.9 ± 0.6
[12]CPP	912	349.1 ± 0.1	393.4 ± 0.6
[6]MCPP	528	215.0 ± 0.2	224.2 ± 0.4
[6,6]CNB	600	229.1 ± 0.5	244.7 ± 0.1

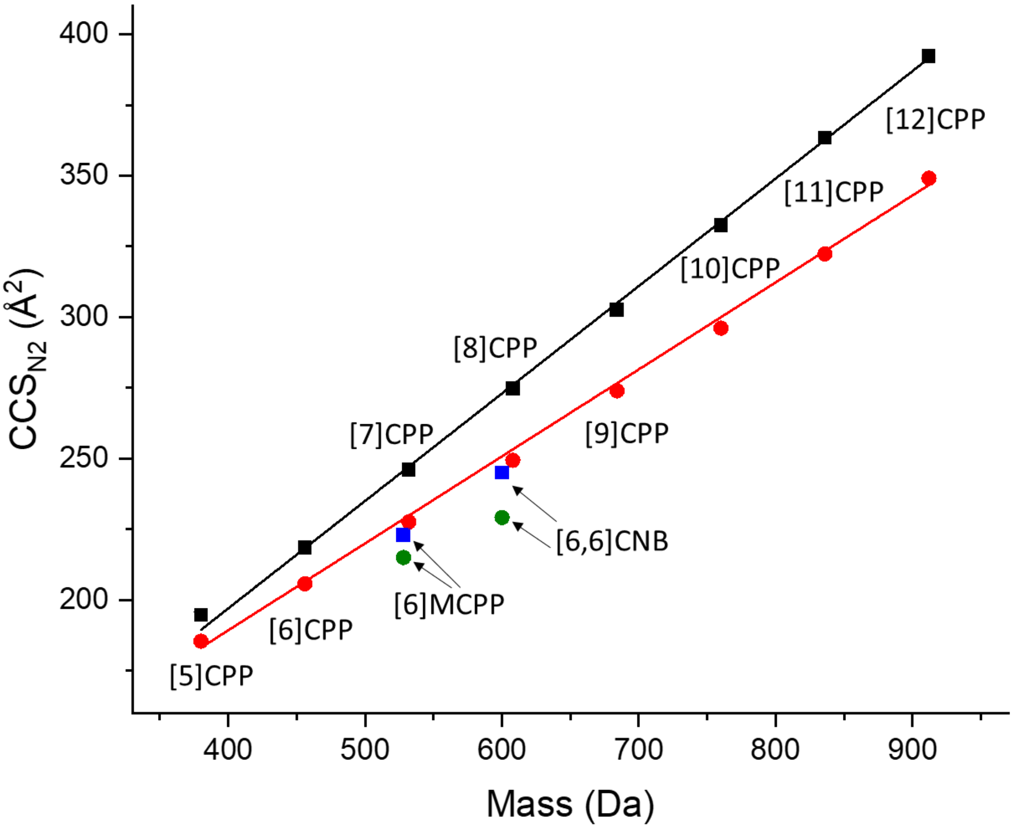

Figure S2: Experimental and theoretical CCS_{N2} values of the isolated carbon nanohoop radical cations in dependence of their mass. Error bars are shown but are smaller than symbol size. Circle: Experimental; Square: Theoretical; Black/Red: Regular [n]CPPs; Blue/Green: [6]MCPP and [6,6]CNB. The experimental and theoretical data for the regular [n]CPPs were fitted and yielded $CCS_{N2} = 66.28855 + 0.30754 \cdot Mass$ (experimental; $R^2 = 0.99899$) and $CCS_{N2} = 45.07573 + 0.37995 \cdot Mass$ (theoretical; $R^2 = 0.99940$).

The agreement between theoretical $^{TH}CCS_{N2}$ and experimental CCS_{N2} values of the regular [n]CPPs as well as [6,6]CNB and [6]MCPP lies between 3 and 12% with the theoretical values being larger. This behaviour is typical for supramolecular complexes and we have previously discussed possible explanations.[15] Both the experimental and theoretical data in the CCS_{N2} vs. *m* give a distinct trend line, which is indicative of a highly similar packing density for the [n]CPPs as discussed in the main text. Interestingly, both datasets yield great fit lines with high R^2 values, but their slopes are significantly different. This leads to the fact that the discrepancy between theory and experiment is larger for the larger nanohoops, whereas there is high agreement for the small [n]CPPs. The occurrence of significantly different fit slopes in homologous series between experimental and theoretical CCS_{N2} values has been previously observed by us for other systems (unpublished results), and we are currently investigating the origin of this phenomenon. Possible explanations are a lack of suitable

parameters for the trajectory method in IMoS[14] or difficulties with accounting for hollow architectures.

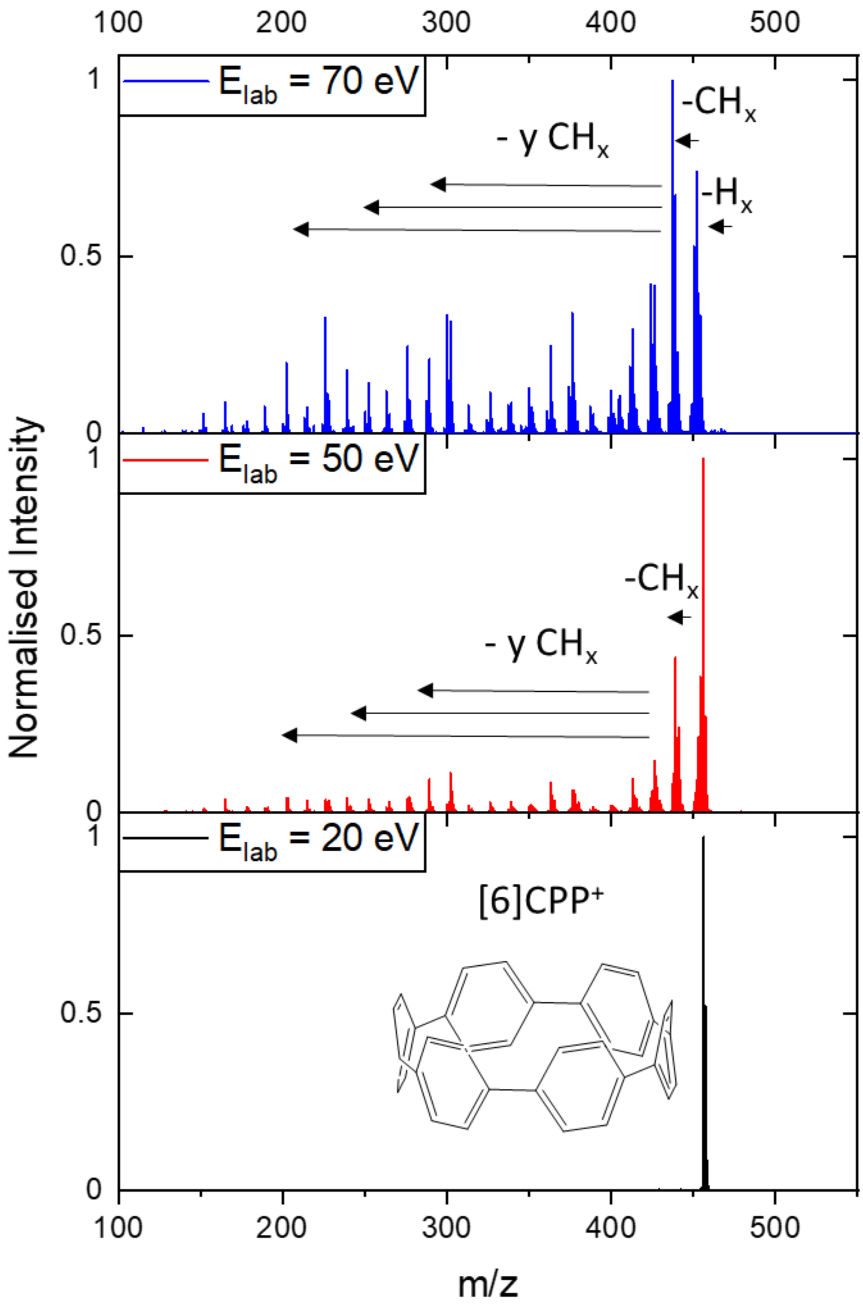

Figure S3: MS^2 spectra of [6]CPP$^+$ at different collision energies. bottom: E_{lab} = 20 eV; centre: E_{lab} = 50 eV; top: E_{lab} = 70 eV. Different fragmentation channels are observed, including the loss of hydrogen atoms and several CH_x units. Precise assignments of fragment ions are difficult as isotopic peaks overlap with those ions that lost different numbers of hydrogens.

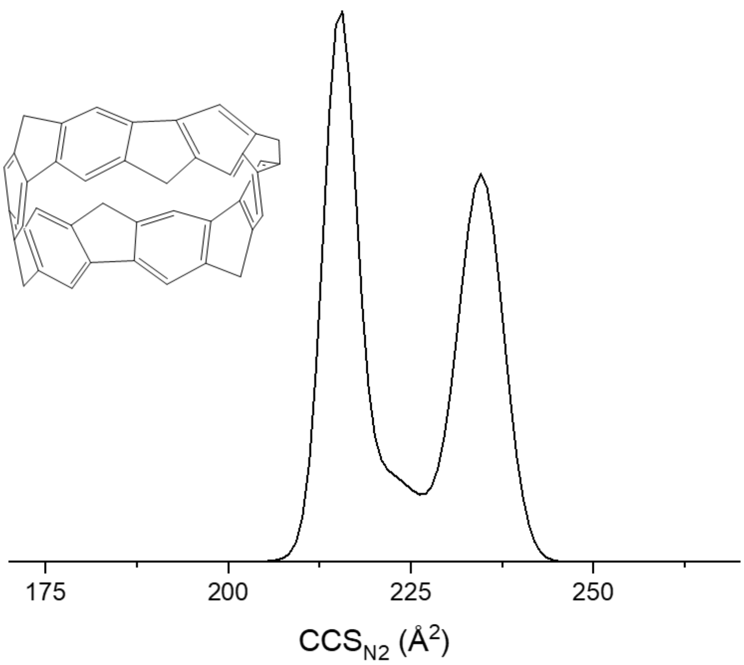

Figure S4: Collision cross section distribution of {[6]MCPP – 2 H}$^+$ at E_{lab} = 80 eV. Two distinct conformations are visible at CCS_{N2} = 216 Å^2 and CCS_{N2} = 235 Å^2 (*m/z* = 526), with the larger one extending beyond CCS_{N2} value of the unactivated precursor ion [6]MCPP$^+$ (CCS_{N2} = 215 Å^2).

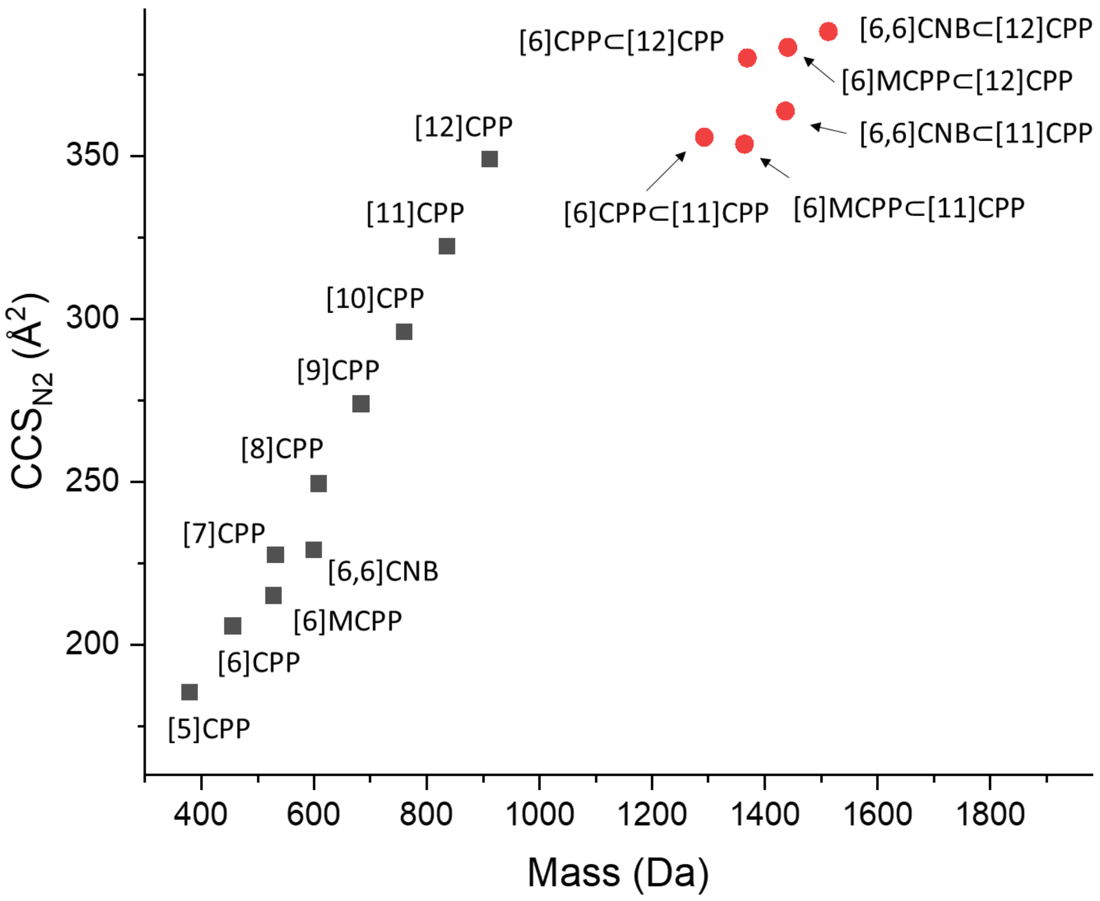

Figure S5: Experimental CCS_{N2} values of the isolated carbon nanohoops and ring-in-ring complexes in dependence of their mass. Error bars are shown but are smaller than symbol size. Red circles: ring-in-ring complexes; black squares: isolated carbon nanohoops. The rings have a significantly steeper slope than the ring-in-ring complexes suggesting that the complexes have a higher packing density as expected.

a)

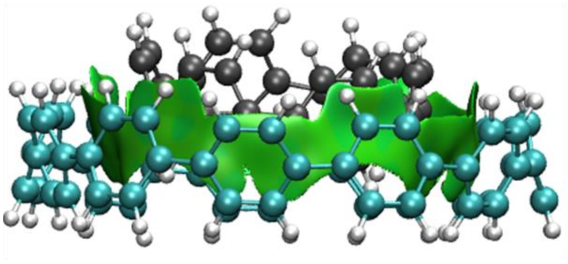

b)

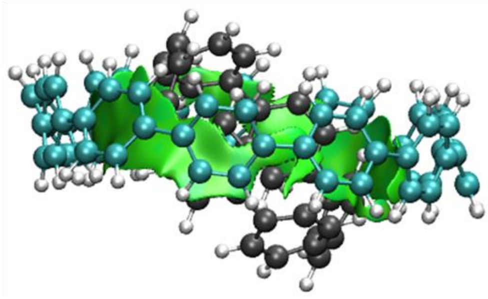

Figure S6: DFT optimised structures of [6]CPP$\subset$[11]CPP with visualization of non-covalent interactions as a) Russian doll and b) planetary orbit. Data from Ref. [3].

a) b)

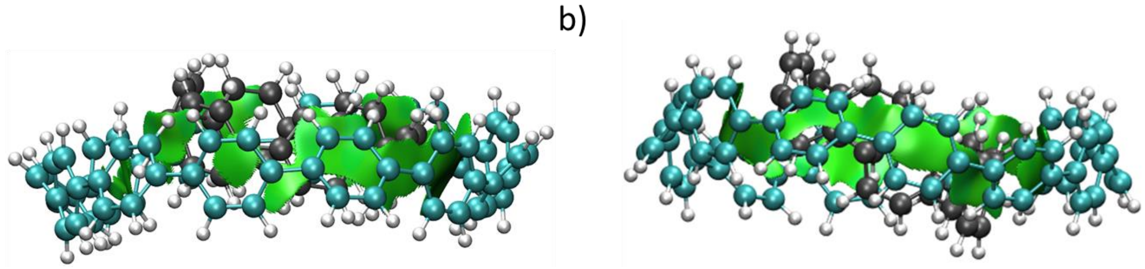

Figure S7: DFT optimised structures of [6]CPP⊂[12]CPP with visualization of non-covalent interactions as a) Russian doll and b) planetary orbit. Data from Ref. [3].

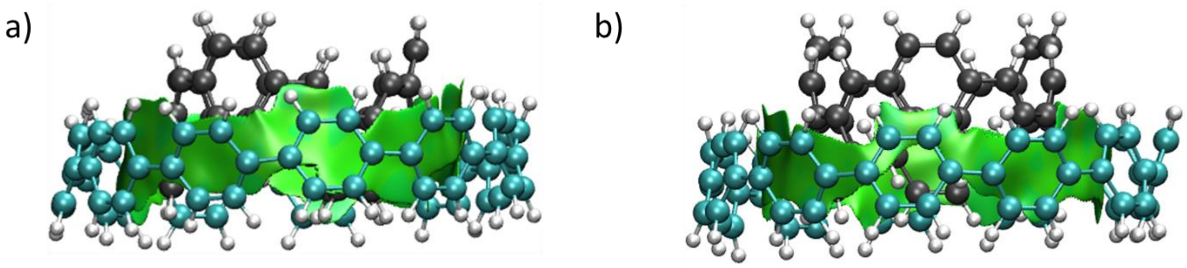

Figure S8: DFT optimised structures of [6,6]CNB⊂[11]CPP with visualization of non-covalent interactions as a) Russian doll and b) planetary orbit (converged to Russian doll). Data from Ref. [3].

a)

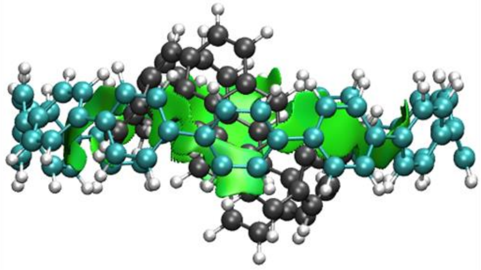

b)

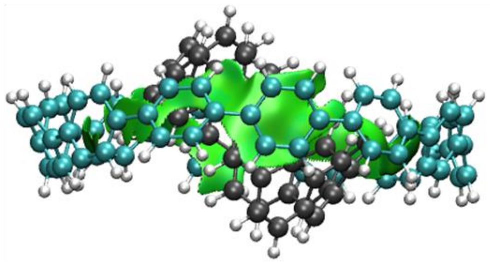

Figure S9: DFT optimised structures of [6,6]CNB⊂[12]CPP with visualization of non-covalent interactions as a) Russian doll (converged to planetary orbit) and b) planetary orbit.

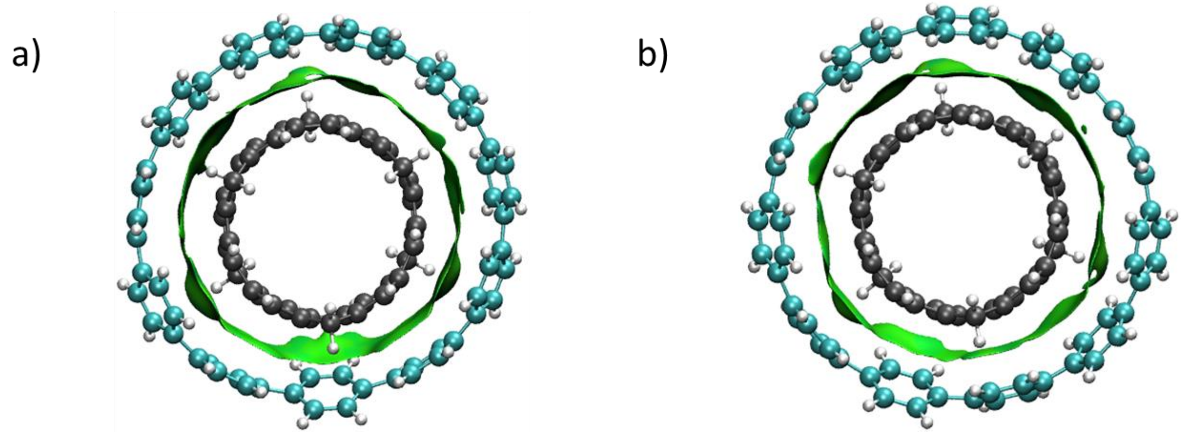

Figure S10: DFT optimised structures of [6]MCPP⊂[11]CPP with visualization of non-covalent interactions as a) Russian doll and b) planetary orbit.

a) b)

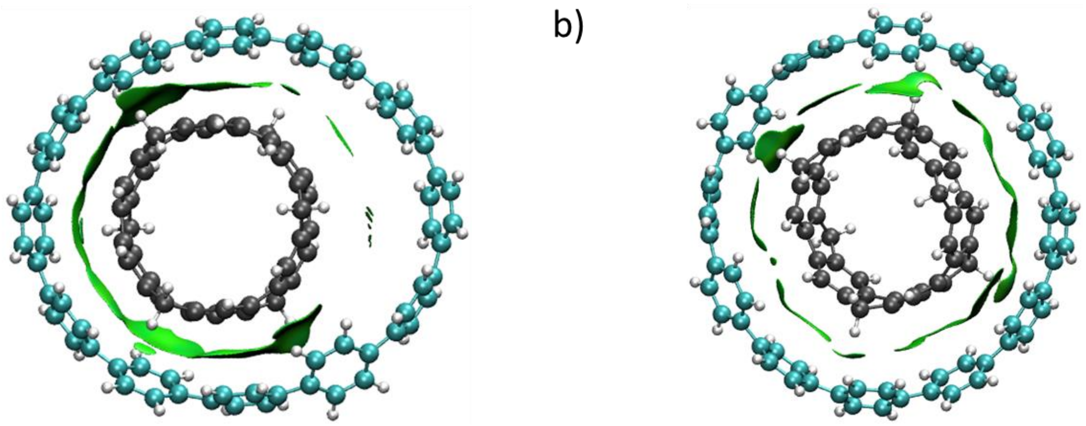

Figure S11: DFT optimised structures of [6]MCPP⊂[12]CPP with visualization of non-covalent interactions as a) Russian doll and b) planetary orbit.

Table S2: Experimental CCS_{N2} values as well as theoretical CCS_{N2} values and fragmentation energies of ring-in-ring complexes in two different conformations (planetary orbit, Russian doll). Fragmentation energies are based on DFT calculations. Data from our previous publication are referenced.[3] *Converged to the other structure.

**Ring-in-ring complex**	**Experimental CCS_{N2} (Å²)**	**$^{TH}CCS_{N2}$ Planetary Orbit (Å²)**	**$^{TH}CCS_{N2}$ Russian Doll (Å²)**	**Fragm. Energy Planetary Orbit (eV)**	**Fragm. Energy Russian Doll (eV)**
[6]CPP⊂[11]CPP	355.8 ± 0.2	399.5 ± 0.3	397.6 ± 1.0	2.03[3]	1.99[3]
[6]CPP⊂[12]CPP	380.2 ± 0.3	435.7 ± 1.0	433.9 ± 1.1	1.71	1.67
[6,6]CNB⊂[11]CPP	363.9 ± 0.6	407.1 ± 0.9	407.9 ± 1.0	2.26*,[3]	2.26[3]
[6,6]CNB⊂[12]CPP	388.3 ± 0.4	450.0 ± 1.3	448.3 ± 0.5	2.00	2.02*
[6]MCPP⊂[11]CPP	353.7 ± 0.7	401.2 ± 1.2	404.1 ± 1.0	2.04	2.11
[6]MCPP⊂[12]CPP	383.4 ± 0.3	441.5 ± 0.7	440.6 ± 1.0	1.74	1.77

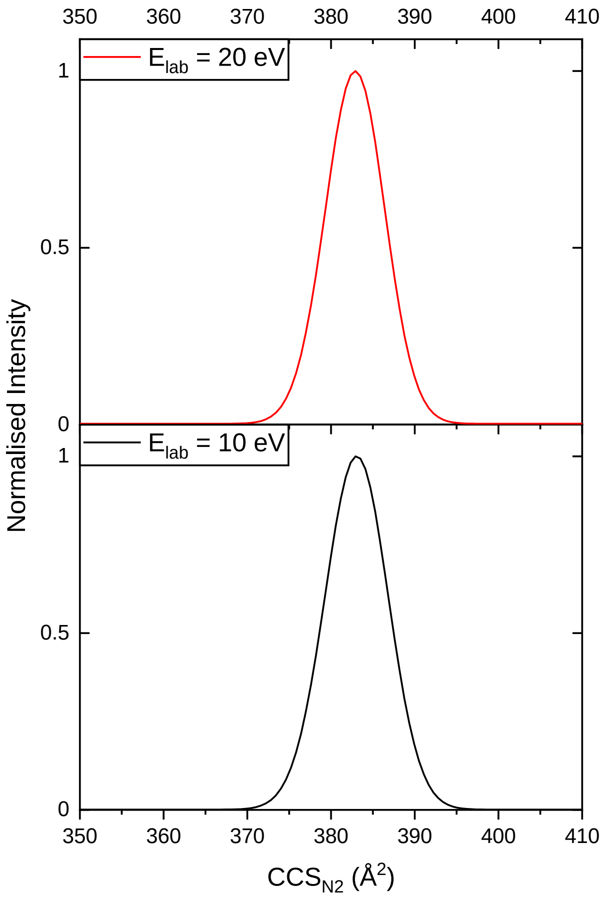

Figure S12: Collision cross section distributions of {[6]MCPP⊂[12]CPP}$^+$ (m/z = 1441) at E_{lab} = 10 eV (bottom) and 20 eV (top). No significant differences were observed.

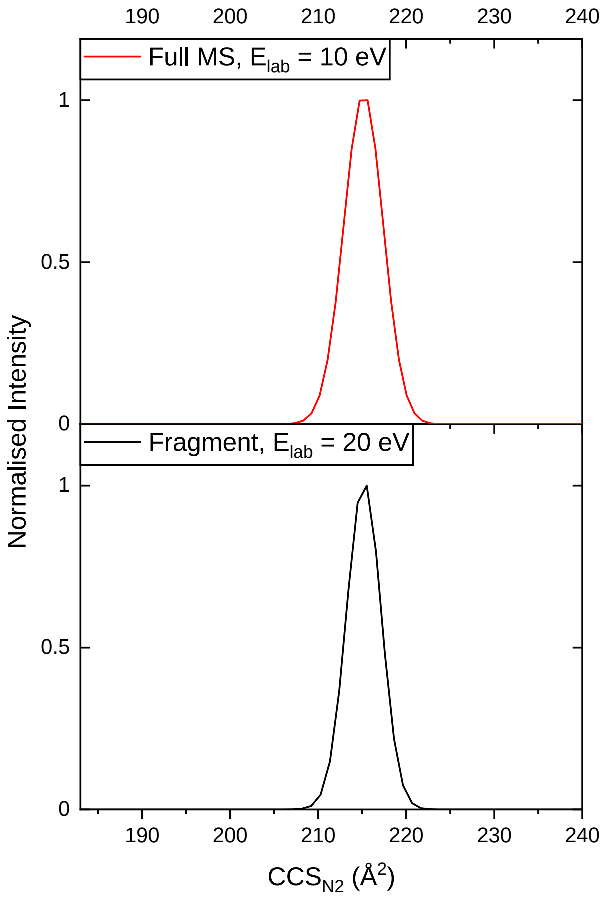

Figure S13: Collision cross section distributions of [6]MCPP$^+$ (m/z = 528); as a fragment of {[6]MCPP⊂[12]CPP}$^+$ at E_{lab} = 20 eV (bottom), and in the full mass spectrum at E_{lab} = 10 eV (top). No significant differences were observed.

References

(1) Ruotolo, B. T.; Benesch, J. L. P.; Sandercock, A. M.; Hyung, S. J.; Robinson, C. V. Ion Mobility-Mass Spectrometry Analysis of Large Protein Complexes. *Nat. Protoc.* **2008**, *3* (7), 1139–1152. https://doi.org/10.1038/nprot.2008.78.

(2) Stow, S. M.; Causon, T. J.; Zheng, X.; Kurulugama, R. T.; Mairinger, T.; May, J. C.; Rennie, E. E.; Baker, E. S.; Smith, R. D.; McLean, J. A.; Hann, S.; Fjeldsted, J. C. An Interlaboratory Evaluation of Drift Tube Ion Mobility-Mass Spectrometry Collision Cross Section Measurements. *Anal. Chem.* **2017**, *89* (17), 9048–9055. https://doi.org/10.1021/acs.analchem.7b01729.

(3) Freiberger, M.; Frühwald, S.; Minameyer, M. B.; Görling, A.; Drewello, T. New Insights into Ring-In-Ring Complexes of [n]Cycloparaphenylenes Including the [12]Carbon Nanobelt. *J. Phys. Chem. A* **2023**, *127* (45), 9495–9501. https://doi.org/10.1021/acs.jpca.3c05644.

(4) Freiberger, M.; Minameyer, M. B.; Solymosi, I.; Frühwald, S.; Krug, M.; Xu, Y.; Hirsch, A.; Clark, T.; Guldi, D. M.; von Delius, M.; Amsharov, K.; Görling, A.; Pérez-Ojeda, M. E.; Drewello, T. Two Rings Around One Ball: Stability and Charge Localization of [1 : 1] and [2 : 1] Complex Ions of [10]CPP and C60/70[*]. *Chem. – Eur. J.* **2023**, *29* (16), e202203734. https://doi.org/10.1002/chem.202203734.

(5) Kertesz, T. M.; Hall, L. H.; Hill, D. W.; Grant, D. F. CE50: Quantifying Collision Induced Dissociation Energy for Small Molecule Characterization and Identification. *J. Am. Soc. Mass Spectrom.* **2009**, *20* (9), 1759–1767. https://doi.org/10.1016/j.jasms.2009.06.002.

(6) Hill, D. W.; Baveghems, C. L.; Albaugh, D. R.; Kormos, T. M.; Lai, S.; Ng, H. K.; Grant, D. F. Correlation of Ecom 50 Values between Mass Spectrometers: Effect of Collision Cell Radiofrequency Voltage on Calculated Survival Yield. *Rapid Commun. Mass Spectrom.* **2012**, *26* (19), 2303–2310. https://doi.org/10.1002/rcm.6353.

(7) *TURBOMOLE | Program Package for Electronic Structure Calculations*. TURBOMOLE. https://www.turbomole.org/ (accessed 2024-03-21).

(8) Becke, A. D. Density-functional Thermochemistry. III. The Role of Exact Exchange. *J. Chem. Phys.* **1993**, *98* (7), 5648–5652. https://doi.org/10.1063/1.464913.

(9) Lee, C.; Yang, W.; Parr, R. G. Development of the Colle-Salvetti Correlation-Energy Formula into a Functional of the Electron Density. *Phys. Rev. B* **1988**, *37* (2), 785–789. https://doi.org/10.1103/PhysRevB.37.785.

(10) Weigend, F.; Ahlrichs, R. Balanced Basis Sets of Split Valence, Triple Zeta Valence and Quadruple Zeta Valence Quality for H to Rn: Design and Assessment of Accuracy. *Phys. Chem. Chem. Phys.* **2005**, *7* (18), 3297. https://doi.org/10.1039/b508541a.

(11) Grimme, S.; Antony, J.; Ehrlich, S.; Krieg, H. A Consistent and Accurate Ab Initio Parametrization of Density Functional Dispersion Correction (DFT-D) for the 94 Elements H-Pu. *J. Chem. Phys.* **2010**, *132* (15), 154104. https://doi.org/10.1063/1.3382344.

(12) Reed, A. E.; Weinstock, R. B.; Weinhold, F. Natural Population Analysis. *J. Chem. Phys.* **1985**, *83* (2), 735–746. https://doi.org/10.1063/1.449486.

(13) Contreras-García, J.; Johnson, E. R.; Keinan, S.; Chaudret, R.; Piquemal, J. P.; Beratan, D. N.; Yang, W. NCIPLOT: A Program for Plotting Noncovalent Interaction Regions. *J. Chem. Theory Comput.* **2011**, *7* (3), 625–632. https://doi.org/10.1021/ct100641a.

(14) Shrivastav, V.; Nahin, M.; Hogan, C. J.; Larriba-Andaluz, C. Benchmark Comparison for a Multi-Processing Ion Mobility Calculator in the Free Molecular Regime. *J. Am. Soc. Mass Spectrom.* **2017**, *28* (8), 1540–1551. https://doi.org/10.1007/s13361-017-1661-8.

(15) Geue, N.; Bennett, T. S.; Arama, A. A.; Ramakers, L. A. I.; Whitehead, G. F. S.; Timco, G. A.; Armentrout, P. B.; Mcinnes, E. J. L.; Burton, N. A.; Winpenny, R. E. P.; Barran, P. E. Disassembly Mechanisms and Energetics of Polymetallic Rings and Rotaxanes. *J. Am. Chem. Soc.* **2022**, *144* (49), 22528–22539. https://doi.org/10.1021/jacs.2c07522.
