# Supplementary material for: Conformational Landscapes and Energetics of Carbon Nanohoops and their Ring-in-Ring Complexes
Source: J Phys Chem Lett. 2024 Jun 24;15(26):6805–11. doi: 10.1021/acs.jpclett.4c01270 (PMC11229059; doi:10.1021/acs.jpclett.4c01270)
Supplement: Supplementary file 2 — jz4c01270_si_002.pdf [file jz4c01270_si_002.pdf]

jz-2024-01270y.R1

Name: Peer Review Information for "Conformational Dynamics and Energetics of Carbon Nanohoops and Their Ring-in-Ring Complexes"

First Round of Reviewer Comments

Reviewer: 1

Comments to the Author

This manuscript describes that the combination of IM-MS, MS2 and DFT calculations enables to characterize the dynamics of carbon nanohoops and their host-guest complexes. Their results show that both isolated nanohoops and combined host-guest complexes are highly rigid when collisionally activated, although the rings can fragment to structures of higher CCS than the precursor. The findings of the different guests [6]CPP, [6]MCPP and

(6,6)CNB in ring-in-ring complexes with [11]CPP and [12]CPP showed interesting trends in CCS, packing density, fragmentation energy calculated with DFT and gas-phase stability, informing on the rules for the formation of such complexes based on their size, structure and

interactions.

Carbon nanohoops and nanobelts are molecules emerged in this decade. There are a lot of carbon nanohoops reported so far and their unique properties have been unveiled. In this time, the authors have discovered a new aspect of such molecules and their host-guest complexes by relatively new analytic methods. I think this work would be worth publishing in JPCL after a minor revision. My concerns are summarized below.

· [6,6]CNB should be (6,6)CNB.

· Are there any discussion about the host-guest complex using [6]MCPP except for its small size? The electronic properties of [6]CPP, [6]MCPP and (6,6)CNB are critically different. If you can find any aspect of electronic properties of guest, please reflect that into the manuscript.

Reviewer: 2

Comments to the Author

This is a nicely written paper describing mass spectrometric and DFT studies of nested molecular hoops. The paper extends work on similar supramolecular complexes described in an earlier JPCA paper by the Drewello group in which many of the more important conclusions regarding the structures and energetics of the ring-ring complexes contained in the current paper were expounded. The new aspects described in this letter relate to an extended range of guest molecules, and collision cross section measurements using travelling wave ion mobility mass spectrometry. In my view, the most interesting observation is that the nested hoops dissociate to give intact charged hoops, confirming that the nested hoops are indeed non-covalently attached to one another.

In my opinion, there are several points that need to be addressed before publication.

1. In determining E50 values, what is the justification for dividing the collision energy by the number of vibrational degrees of freedom for the host? Why are the guest vibrational degrees of freedom ignored in determining DOF? Surely, there should be efficient flow of vibrational energy between the two rings prior to dissociation. The site of the initial collision is irrelevant – division of the collision energy by DOF is a crude means to account for the sharing of energy between the vibrational degrees of freedom.

In the SI, a previous paper from the Drewello group is cited to justify the approach. However, the cited paper also contains no justification. Indeed, the relevant section in the SIs for the two papers are almost same (word for word), except that the earlier paper does not provide a citation for the "previous results". If this approach is to be used to gauge the relative stabilities of different complexes, it needs to be justified properly. The explanation in the SI section of the paper (and in the previous paper) is inadequate. What is the theoretical justification for the approach? Are there precedents in the literature?

Why is Elab used and not Ecm? In practice it probably doesn't make much difference, except if the collision gas is changed (e.g. He or Ar rather than N2). Actually, if the approach is robust, other collision gases should give the same ordering for the binding energies.

On P10 line 235 it is stated that:" The data show that [11]CPP is the more suitable host for [6]CPP and [6]MCPP, whereas in the case of the nanobelt [6,6]CNB the host [12]CPP is preferred (Table 1)." Actually, the DFT data in Table 1 indicate that for the [6,6]CNB the host [11]CPP is energetically preferred. Indeed, the DFT calculations suggest that all three guests ([6]CPP, [6,6]CNB, [6]MCCP) are bound more strongly to [11]CPP than to [12]CPP. The question is how reliable are the E50 values for estimating relative stabilities given the rather arbitrary definition for DOF.

2. In several places in the paper it is claimed that the nanohoops and the complexes are "highly rigid". I guess it depends on the definition of "rigidity". CCS measurements can be completely insensitive to structural deformation if that deformation does not lead to a change in the CCS. Indeed, the CPPs should be extremely fluxional for motions involving internal rotation of the phenyl rings about the connecting bonds in the para positions. As well, the rings should be easy to squash such that when viewed from above they would appear as ellipses rather than circles. Examination of the vibrational modes and frequencies obtained from DFT calculations would allow the "soft" modes of the complexes to be identified. In most instances in the paper "structural rigidity" should be replaced by "structural integrity" – there are no irreversible changes in structure due to the breaking of covalent or non-covalent bonds.

3. Collisional activation of the [6]CPP+ hoop at an energy of 55 eV, results in structural changes and the appearance of two new peaks in the mobility distribution (Figure 3(c)). One can imagine that opening of the hoop would be most easily accomplished by breaking a C-C bond linking phenyl rings. Why would there be two peaks for ring-open forms? Were structures calculated for possible ring-open forms? "Conformational landscape" is often used in the paper, but few conformations are calculated, especially for the fragments.

4. One of the interesting aspects of the fragmentation of the rings is that the smaller guest ring carries the positive charge. One would expect that the fragment ring with lower IP would carry the positive charge. What are the calculated ionization potentials of the various rings? The calculations in ref. 37 indicate that the IP is almost independent of ring size, with n=5 and 6 having IPs that are around 0.1 eV less than n=11 and 12. Is it possible that CID of the complexes involves, opening of the outer ring liberating a charged inner ring, given that the calculated IPs for the ring-open isomers are higher than for the hoops (ref. 37 Table 2)?

Other points

1. Regarding the title of the paper, is any of the work concerned with "Conformational dynamics"? Conformations and energetics, yes, but where does the paper deal with dynamics? Neither the experiments nor the calculations are concerned with the time dependence of structures.

2. From the difference in the sizes of the host and guest one can estimate the mean separation between the rings. For example, for the CPP hoops, a difference of 5 phenyl units would correspond to a difference in the radii of the two rings of $5 \times 3 \times 1.3$Å$/(2\pi) = 3.1$Å, assuming a C-C bond length of 1.3 Å. How would the difference in radii compare with the separation between two benzene molecules in the benzene dimer in a slipped parallel arrangement?

Author's Response to Peer Review Comments:

see attached. We have addressed all comments and include a track changed version of the new submission for review only

Response to Decision Letter

Dear Editor,

We thank you and the reviewers for their careful evaluation of our manuscript and for the insightful suggestions to improve the paper. We believe we have fully answered the reviewers' comments and have included a track changed and clean version as requested. Please find the answers to specific points below.

We have further adjusted the format of the manuscript as requested.

Reviewer(s)' Comments to Author:

Reviewer: 1

Recommendation: This paper is publishable subject to minor revisions noted. Further review is not needed.

Comments:
This manuscript describes that the combination of IM-MS, MS2 and DFT calculations enables to characterize the dynamics of carbon nanohoops and their host-guest complexes. Their results show that both isolated nanohoops and combined host-guest complexes are highly rigid when collisionally activated, although the rings can fragment to structures of higher CCS than the precursor. The findings of the different guests [6]CPP, [6]MCPP and (6,6)CNB in ring-in-ring complexes with [11]CPP and [12]CPP showed interesting trends in CCS, packing density, fragmentation energy calculated with DFT and gas-phase stability, informing on the rules for the formation of such complexes based on their size, structure and interactions.

Carbon nanohoops and nanobelts are molecules emerged in this decade. There are a lot of carbon nanohoops reported so far and their unique properties have been unveiled. In this time, the authors have discovered a new aspect of such molecules and their host-guest complexes by relatively new analytic methods. I think this work would be worth publishing in JPCL after a minor revision. My concerns are summarized below.

We thank the reviewer for their positive comments and the opportunity to revise our manuscript.

[6,6]CNB should be (6,6)CNB.

We believe that this nomenclature is acceptable as it is, and would prefer keeping it consistently for [6]CPP, [6]MCPP and [6,6]CNB, although their structures are differently related to their names/abbreviations. The nomenclature of these nanohoops in the literature is in general not consistent and ours has been previously used in other publications (e.g in DOI: 10.1088/2053-1591/ac7382).

Are there any discussion about the host-guest complex using [6]MCPP except for its small size? The electronic properties of [6]CPP, [6]MCPP and (6,6)CNB are critically different. If you can find any aspect of electronic properties of guest, please reflect that into the manuscript.

Itami and co-workers have calculated the HOMO and LUMO levels of [6]MCPP in comparison to [6]CPP, showing that the HOMO of [6]MCPP is slightly higher than that of [6]CPP (DOI:

10.1021/jacs.0c06007). We are not aware that this difference has an impact on the π-π interactions within the host-guest complexes and hence their stabilities. As indicated in the manuscript, we believe that the observed trends are a function of ring rigidity as well as host/guest size differences, which in turn influence the overlap of the π-orbitals between both species. We suggest that, while interesting, further discussions on the impact of the electronic guest properties are beyond the scope of this letter, but this work may inspire future studies of that nature.

Reviewer: 2

Recommendation: This paper may be publishable, but major revision is needed; I would like to be invited to review any future revision.

Comments:
This is a nicely written paper describing mass spectrometric and DFT studies of nested molecular hoops. The paper extends work on similar supramolecular complexes described in an earlier JPCA paper by the Drewello group in which many of the more important conclusions regarding the structures and energetics of the ring-ring complexes contained in the current paper were expounded. The new aspects described in this letter relate to an extended range of guest molecules, and collision cross section measurements using travelling wave ion mobility mass spectrometry. In my view, the most interesting observation is that the nested hoops dissociate to give intact charged hoops, confirming that the nested hoops are indeed non-covalently attached to one another.

In my opinion, there are several points that need to be addressed before publication.

We thank the reviewer for their positive evaluation of our paper and have addressed their concerns below.

1. In determining E50 values, what is the justification for dividing the collision energy by the number of vibrational degrees of freedom for the host? Why are the guest vibrational degrees of freedom ignored in determining DOF? Surely, there should be efficient flow of vibrational energy between the two rings prior to dissociation. The site of the initial collision is irrelevant – division of the collision energy by DOF is a crude means to account for the sharing of energy between the vibrational degrees of freedom. In the SI, a previous paper from the Drewello group is cited to justify the approach. However, the cited paper also contains no justification. Indeed, the relevant section in the SIs for the two papers are almost same (word for word), except that the earlier paper does not provide a citation for the "previous results". If this approach is to be used to gauge the relative stabilities of different complexes, it needs to be justified properly. The explanation in the SI section of the paper (and in the previous paper) is inadequate. What is the theoretical justification for the approach? Are there precedents in the literature?

The justification for choosing this less conventional energetic scale is found in a previous publication (DOI: 10.1002/chem.202203734), and we have now added this citation to the supporting information of the manuscript under review. We apologise for this oversight. The supporting information of DOI: 10.1002/chem.202203734 also contains several other references that demonstrate the suitability of the energy scale E_{lab}/DoF. Briefly, we suggest that the guest is largely shielded from direct collisions with the inert gas, and that the energy transfer between the host and guest is more difficult than for covalently bound compounds (based on an example of nanohoop fullerene complexes). For the present case of ring-in-ring host guest complex, the vibrational excitement of the guest is further hindered as its strain energy is significantly larger than that of the host (DOI: 10.1021/accountsmr.1c00105). Vibrational activation of a more strained guest requires

more energy than the activation of the less strained host, and enhanced movement of the host compared to the guest seems reasonable.

We have also compared the E_{50} values based on E_{com} with those in the manuscript that are based on E_{lab} divided by DoF, and we found the same qualitative trends (Table R1, for Reviewers only). As the scope of this letter are the properties of the ring-in-ring complexes, and considering that the energy scale does not cause a difference in the trends, we believe that our rationale is sufficient. As mentioned, we have now referenced our previous publication containing the detailed justification.

Table R1 (for Reviewers only): Comparison of E_{50} values based on E_{com} and E_{lab}/DoF for all discussed ring-in-ring complexes.

Ring-in-ring complex	E_{50} Values (E_{com})	E_{50} Values (E_{lab}/DoF (CPP))
[6]CPP⊂[11]CPP	0.45	0.066
[6]CPP⊂[12]CPP	0.45	0.064
[6,6]CNB⊂[11]CPP	0.48	0.078
[6,6]CNB⊂[12]CPP	0.57	0.088
[6]MCPP⊂[11]CPP	0.53	0.081
[6]MCPP⊂[12]CPP	0.50	0.074

Why is Elab used and not Ecm? In practice it probably doesn't make much difference, except if the collision gas is changed (e.g. He or Ar rather than N2). Actually, if the approach is robust, other collision gases should give the same ordering for the binding energies.

E_{com} provides a physically correct description for the transfer of kinetic energy into internal energy for a single collision, however multiple collisions occur in commercial CID cells. As stated above, several literature precedents (as cited in the SI of DOI: 10.1002/chem.202203734) suggest that this can alternatively be accounted for when dividing E_{lab} by DoF. Hence, dividing E_{com} by DoF would lead to a double correction of the same phenomenon, and is hence not advisable.

On P10 line 235 it is stated that: "The data show that [11]CPP is the more suitable host for [6]CPP and [6]MCPP, whereas in the case of the nanobelt [6,6]CNB the host [12]CPP is preferred (Table 1)." Actually, the DFT data in Table 1 indicate that for the [6,6]CNB the host [11]CPP is energetically preferred. Indeed, the DFT calculations suggest that all three guests ([6]CPP, [6,6]CNB, [6]MCCP) are bound more strongly to [11]CPP than to [12]CPP. The question is how reliable are the E50 values for estimating relative stabilities given the rather arbitrary definition for DOF.

The reviewer is correct stating that the DFT fragmentation energy trends of the [6,6]CNB complexes are different to those obtained experimentally as E_{50} values, however we have shown above that our definition of E_{50} is not arbitrary and that the trends agree with those of more conventional E_{50} definitions with E_{com} (Table R1). We transparently included both experimental and computational

stability data in the manuscript, and the reason for the disagreement in this single case is not obvious. We note other trends are well reproduced by the DFT calculations.

2. In several places in the paper it is claimed that the nanohoops and the complexes are "highly rigid". I guess it depends on the definition of "rigidity". CCS measurements can be completely insensitive to structural deformation if that deformation does not lead to a change in the CCS. Indeed, the CPPs should be extremely fluxional for motions involving internal rotation of the phenyl rings about the connecting bonds in the para positions. As well, the rings should be easy to squash such that when viewed from above they would appear as ellipses rather than circles. Examination of the vibrational modes and frequencies obtained from DFT calculations would allow the "soft" modes of the complexes to be identified. In most instances in the paper "structural rigidity" should be replaced by "structural integrity" – there are no irreversible changes in structure due to the breaking of covalent or non-covalent bonds.

We agree with the reviewer that the discussed nanohoops are not rigid in a way that implies no structural changes, and we have previously published molecular dynamics simulations of a host-guest complex between [10]CPP and a C_{70} fullerene (DOI: 10.1002/chem.202203734, Figure S16) showing the exact behaviour of "squashing" in CPPs that the reviewer suggested. Although ion mobility as a technique is highly sensitive towards minor structural changes and our data suggests that the ring structure is overall maintained, we agree with the reviewer and have changed the wording "structural rigidity" to "structural integrity" throughout the manuscript.

3. Collisional activation of the [6]CPP+ hoop at an energy of 55 eV, results in structural changes and the appearance of two new peaks in the mobility distribution (Figure 3(c)). One can imagine that opening of the hoop would be most easily accomplished by breaking a C-C bond linking phenyl rings. Why would there be two peaks for ring-open forms? Were structures calculated for possible ring-open forms? "Conformational landscape" is often used in the paper, but few conformations are calculated, especially for the fragments.

The fragmentation pattern of [6]CPP$^+$ indicates that there will be extensive isomerization upon ring opening, and we agree with the reviewer that the C-C bonds between the phenyl units are the most likely locations of cleavage in the nanohoops. This likely results in the conformations with higher CCS_{N2} values, and this applies both for the collisionally activated precursor ion (Figure 3c) and the fragment in Figure 3d. Myriad structures and conformations of both the precursor ion (m/z = 456) and the discussed fragment (m/z = 454) are possible, also including the opening of benzene units. While further calculations on the fragmentation process are in principle possible, they are non-trivial and at least partially speculative, and we suggest that these are not within the scope of this letter.

4. One of the interesting aspects of the fragmentation of the rings is that the smaller guest ring carries the positive charge. One would expect that the fragment ring with lower IP would carry the positive charge. What are the calculated ionization potentials of the various rings? The calculations in ref. 37 indicate that the IP is almost independent of ring size, with n=5 and 6 having IPs that are around 0.1 eV less than n=11 and 12. Is it possible that CID of the complexes involves, opening of the outer ring liberating a charged inner ring, given that the calculated IPs for the ring-open isomers are higher than for the hoops (ref. 37 Table 2)?

Using tandem mass spectrometry, we have previously shown that the guest molecules retain the charge after collisional dissociation of the ring-in-ring complexes (DOI: 10.1021/acs.jpca.3c05644). This agrees with the study of Yamago and co-workers, who have used cyclic voltammetry to

investigate the ionization potentials of [5 –12]CPPs, showing that small CPPs exhibit a significantly lower oxidation potential than large CPPs (DOI: 10.1002/asia.201600582). Hence, there is strong experimental evidence from both cyclovoltammetry and tandem mass spectrometry for the fact that the smaller guest nanohoops retain the charge. We have indeed calculated the ionization potentials of the rings, but have not included them in the manuscript as they do not match our and other groups experimental observations. This is not surprising, as modern DFT functionals with in-built dispersion correction are good in estimating noncovalent bond strengths, but not necessarily correct in determining molecular characteristics such as ionization potentials.

We do not believe the opening of the outer ring is necessary for the loss of the guest loss, as this would result in a significant precursor ion peak at higher CCS_{N2} values. Although Figure 3c shows enlarged species of the same mass, they do only occur as minor species and can hence not explain an open ring structure as a necessity for losing the inner nanohoop, which occurs almost exclusively as shown in the MS^2 spectra (e.g Figure 4).

Other points:

Regarding the title of the paper, is any of the work concerned with "Conformational dynamics"? Conformations and energetics, yes, but where does the paper deal with dynamics? Neither the experiments nor the calculations are concerned with the time dependence of structures.

We agree with the reviewer and have changed the title to "Conformational Landscapes and Energetics of Carbon Nanohoops and their Ring-In-Ring Complexes".

From the difference in the sizes of the host and guest one can estimate the mean separation between the rings. For example, for the CPP hoops, a difference of 5 phenyl units would correspond to a difference in the radii of the two rings of 5x3x1.3Å/(2π)=3.1Å, assuming a C-C bond length of 1.3 Å. How would the difference in radii compare with the separation between two benzene molecules in the benzene dimer in a slipped parallel arrangement?

This is an intriguing question that has been discussed previously by Bachrach and Zayat (DOI: 10.1021/acs.joc.6b00339). Their calculations show that the distance between two CPPs is ca. 3.4 Å for a ring size difference of five phenyl units in the host-guest complexes, which agrees with the distance between two benzene rings in a slipped parallel dimer arrangement (r = 3.37 Å) as shown in the same paper.

Additional Reviewer Comment:

On the whole this is a nice paper (it could have been even nicer if measured with Cyclic-IMS which the Barran group also has - but this is only a minor point).

We thank the reviewer for their positive evaluation, and we will attempt to investigate the conformational landscapes of such species in future work with our Cyclic-IMS instrument and other forms of ion mobility apparatus.

My only substantive comment is that it would help the reader if all CCS numbers (theo and exp) which are now distributed throughout fig2, figS2, figS5 and table 1, tableS1, tableS2 could be collected in one table.

We have now added the experimental *CCS_{N2}* values of the ring-in-ring complexes to Table S2. Now all the *CCS_{N2}* values are contained in Table S1 (for the isolated nanohoops) and Table S2 (for the ring-in-ring complexes). We would prefer to keep the data tables divided between rings and ring-in-ring complexes, in order to make it more accessible regarding the section of the manuscript that is referred to.

jz-2024-01270y.R2

Name: Peer Review Information for "Conformational Landscapes and Energetics of Carbon Nanohoops and their Ring-In-Ring Complexes"

Second Round of Reviewer Comments

Reviewer: 2

Comments to the Author

For the most part the authors have adequately addressed the concerns raised in my initial review and in my opinion the paper is now suitable for publication.

Notwithstanding this recommendation, I am still unconvinced by the assumption that the inner hoop is shielded from collisions and that its DOF should not be included when determining E50. As pointed out in the initial review, the vibrational modes of the two hoops should be coupled. For example, one can imagine that squashing the outer hoop, will lead to the inner hoop also being squashed. Furthermore, most of the inner hoop's vibrational modes will have very similar frequencies to those of the outer hoop, facilitating near-resonant vibrational energy transfer.

Author's Response to Peer Review Comments:

thank you, we have made these changes.
